# Understanding geographic and racial/ethnic disparities in mortality from four major cancers in the state of Georgia: a spatial epidemiologic analysis, 1999–2019

**DOI:** 10.1038/s41598-022-18374-7

**Published:** 2022-08-19

**Authors:** Justin Xavier Moore, Martha S. Tingen, Steven S. Coughlin, Christine O’Meara, Lorriane Odhiambo, Marlo Vernon, Samantha Jones, Robert Petcu, Ryan Johnson, K. M. Islam, Darryl Nettles, Ghadeer Albashir, Jorge Cortes

**Affiliations:** 1 Department of Medicine, Georgia Prevention Institute, Cancer Prevention, Control, & Population Health, Medical College of Georgia, Georgia Cancer Center, Augusta University, 1410 Laney Walker Blvd CN-2120, Augusta, GA 30912 USA; 2 Institute of Preventive and Public Health, Medical College of Georgia, Augusta University, Augusta, GA USA; 3 Department of Population Health Sciences, Medical College of Georgia, Augusta University, Augusta, GA USA; 4 Georgia Cancer Center, Augusta University, Augusta, GA USA

**Keywords:** Epidemiology, Breast cancer, Cancer epidemiology, Lung cancer, Colorectal cancer, Prostate cancer

## Abstract

We examined geographic and racial variation in cancer mortality within the state of Georgia, and investigated the correlation between the observed spatial differences and county-level characteristics. We analyzed county-level cancer mortality data collected by the Centers for Disease Control and Prevention on breast, colorectal, lung, and prostate cancer mortality among adults (aged ≥ 18 years) in 159 Georgia counties from years 1999 through 2019. Geospatial methods were applied, and we identified hot spot counties based on cancer mortality rates overall and stratified by non-Hispanic white (NH-white) and NH-black race/ethnicity. Among all adults, 5.0% (8 of 159), 8.2% (13 of 159), 5.0% (8 of 159), and 6.9% (11 of 159) of Georgia counties were estimated hot spots for breast cancer, colorectal, lung, and prostate cancer mortality, respectively. Cancer mortality hot spots were heavily concentrated in three major areas: (1) eastern Piedmont to Coastal Plain regions, (2) southwestern rural Georgia area, or (3) northern-most rural Georgia. Overall, hot spot counties generally had higher proportion of NH-black adults, older adult population, greater poverty, and more rurality. In Georgia, targeted cancer prevention strategies and allocation of health resources are needed in counties with elevated cancer mortality rates, focusing on interventions suitable for NH-black race/ethnicity, low-income, and rural residents.

## Introduction

Cancer is the second leading cause of morbidity and mortality in the United States (U.S.), responsible for an estimated 1.9 million new cases and 609,360 deaths in 2022^1^. Specifically among men, cancers of the lung, prostate, and colon and rectum (i.e., colorectal) account for 46% and 42% of all incident cancer cases and deaths, respectively^2^. Similarly among women, lung, breast, and colorectal cancers account for about 50% and 45% of all incident cases and cancer deaths, respectively^2^. In the general population, cancer mortality has decreased by nearly 31% since its apex in 1991, due in part to tobacco control and prevention strategies such as smoking cessation and smoke-free policies. The advent of modern cancer detection, screening, and therapeutics including targeted chemotherapy, surgical precision, and medications also contributed to the decline^2^.

However, specific populations such as NH-black adults, and those living in communities with higher poverty rates (including rural communities) have not shared equally in improvements in cancer mortality and continue to experience lower relative survival^3–5^. Further, counties in the southeastern United States have experienced an increased burden in mortality for all cancer sites combined from 1980 through 2014^6^. NH-black adults suffer from the highest mortality rates of all cancer sites combined in the United States and in Georgia^5,7^. Mortality-to-incidence ratios are higher for NH-black adults for each cancer site when compared to NH-white adults^5,7^. For example, compared to NH-white adults, 5-year survival for colorectal cancer was 20% lower in NH-black adults (17.2% vs. 21.5%)^8^. In Georgia, female breast cancer, prostate cancer, lung cancer, and colorectal cancer comprise the top four cancers in terms of incidence and mortality^2^. Female breast cancer has the highest incidence (126.8 per 100,000) while lung cancer has the highest mortality (40.9 per 100,000)^2^. Racial differences in late-stage diagnosis and survival persist for NH-black adults when compared with NH-white adults^9–13^. The intersectionality between rurality and race/ethnicity plays a significant role in cancer risk and survival. Zahnd et al.^14^ reported that compared to patients residing in urban areas, rural residents were at a 5% increased risk of distant stage diagnoses of all stageable cancers (relative risk 1.05, 95% CI: 1.05–1.06); and specifically among rural populations, NH-black adults were at a 13% increased risk of distant stage diagnoses of all stageable cancers when compared to NH-whites (relative risk 1.13, 95% CI: 1.12–1.15)^14^.

While several studies have examined the effects of race and ethnicity and/or geography on cancer mortality, very few studies have explored the independent and joint effects of geography and race on multiple cancer mortality for the state of Georgia^15^. Racial and socioeconomic characteristics vary greatly from region to region, making the specific analysis of individual states relevant as they may provide more granular information^16,17^. Identifying cancer hotspots in Georgia stratified by race/ethnicity can be useful in allocating resources for prevention and early treatment of the most prevalent cancer types. The findings from this study may help understand social determinants of health specific for Georgia communities regarding cancer mortality prevention and control. Furthermore, understanding the current racial and geographic distribution of cancer mortality is the first step in attempts to mitigate health disparities in Georgia. In this study, we aimed to identify significant clusters of higher cancer mortality for cancers of breast, colorectal, lung, and prostate, and examine whether these areas of high-risk are modified by race/ethnicity. We hypothesize that counties with higher density of racial/ethnic minority populations living in rural communities may have the greatest burden of cancer mortality, and thus highlight as ‘hot spots’ for various cancer mortality.

## Methods

### Ethical statement

We utilized pre-existing secondary data that are publicly available and non-identifiable; thus, this study was considered exempt by the Institutional Review Board of Augusta University. All experiments were performed in accordance with relevant guidelines and regulations.

### Study design and population

We performed an ecologic analysis among U.S. adults aged 18 + residing within the state of Georgia from 1999 through 2019. We elected this 20-year period to (1) provide robust estimation of county-level measures of hotspots even when stratified by race/ethnicity, and (2) to delineate areas with persistent burden of cancer mortality. We utilized county-level cancer mortality (breast, colon and rectum, lung, and prostate) collected by the Centers for Disease Control and Prevention (CDC) and National Center for Health Statistics (NCHS). We further linked this county-level mortality data with their corresponding county-level characteristics from the 2020 County Health Rankings (CHR) data.

### Primary outcomes & identification of cancer deaths

We obtained cancer specific mortality for years 1999 through 2019 from cancers of the breast, colorectal, lung, and prostate at the county-level using the CDC, NCHS, and the Underlying Cause of Death online database (https://wonder.cdc.gov)^18^. The underlying cause-of-death defines attributable deaths as “the disease or injury which initiated the train of events leading directly to death, or the circumstances of the accident or violence which produced the fatal injury”^18^. Data from the underlying cause-of-death are derived from death certificates and include a record for every death of a U.S. resident. We identified the county-level totals, crude rates, and age-adjusted rates for each cancer using the following ICD-10 codes: breast (C50.0 through C50.9), colon (C18.0 through C18.9), rectosigmoid junction (C19), rectum (C20), lung and bronchus (C34.0 through C34.9), and prostate (C61). Population estimates were generated for those aged 18 and older for each Georgia county for the years 1999 through 2019 using intercensal estimates for non-census years. We obtained the mortality rate per 100,000 population (per 100,000 women population for breast cancer, and per 100,000 men population for prostate cancer) using these county-level populations years 1999 through 2019 as the denominator. We identified cancer-specific deaths overall and by race/ethnicity for NH-black and NH-white adults.

### County-level community health characteristics

We linked the county-level data on cancer mortality with county-level data on sociodemographic and social determinants of health factors. We obtained county-level characteristics from the 2020 County Health Rankings (CHR)^19,20^ including race/ethnicity, sex, age, adult obesity, adult tobacco smoking prevalence, adults with some college education, median household income, proportion of population with limited access to healthy foods, primary care physicians per 10,000 population, and proportion of population living in rural areas. CHR consists of nationally representative data collected from a sample of the total non-institutionalized population over 18 years of age living in households. Detailed descriptions of county-level characteristics are described in Supplemental Table 1. Using estimates from the 2020 CHR, we presented data on county-level proportions of race/ethnic groups for the four most populous groups in Georgia for the 20-year period including: NH-white, NH-black, Hispanic or Latinx, and Asian. Proportion of rural county-level residents was defined based on 2010 Rural–Urban Commuting Areas (RUCA) classifications. The 10 RUCA codes were aggregated into a dichotomized variable: (1) Urban (i.e., population centers with 50,000 or more residents) and (2) Non-Urban (i.e., towns or small cities with population centers with less than 50,000 residents), and proportions were determined by 2020 CHR^21,22^.

### Geospatial analysis & cancer hot spots derivation

Geospatial hot spots for diseases or prevalence of conditions are explained as the spatial collection of cases in a specific subpopulation^23^. To date, there is no formal and/or gold standard for identifying spatial disease clustering. However, there are several geospatial measures that account for overall rate, county population, and spatial correlation for geographic areas of interest^24,25^. Further, ‘hot spots’ indicate geographic areas of higher disease burden. To identify hot spots for breast, colorectal, lung, and prostate cancer mortality, we derived county-level estimates of high-risk and geographic clustering of cancer mortality using methodology previously described by Moore et al. ^24,25^. We performed three geospatial autocorrelation methods: empirical Bayes (EB) smoothed mortality rates^26^; local Moran’s I with EB rate which is known as local indicators of spatial association (LISA)^27^; and the Getis-Ord Gi* statistic^28,29^. In Supplemental Material 1 we provide detailed information regarding our geospatial methodology.

We categorized counties as “hot spots” for each cancer-specific mortality (and when stratified by race/ethnicity) if they were estimated (1) within the fifth quintile of smoothed spatial Empirical Bayes (EB) cancer mortality rates, and either a 2) high-high cluster using Local Indicators of Spatial Association (LISA), or 3) determined as a hot-spot by Getis-Ord Gi* statistic^26–31^. All other Georgia counties were categorized as non-hot spots. We performed all geospatial analyses using GeoDa version 1.16.0.16^32^ with 99,999 permutations and random seed number 74, when necessary (LISA and Gi* analyses). All mapping were performed using ArcGIS 10.7^33^. We provided lists of Georgia counties and their hot spot designation overall, and by race for breast cancer mortality (Supplemental Table 2), colorectal cancer mortality (Supplemental Table 3), lung cancer mortality (Supplemental Table 4), and prostate cancer mortality (Supplemental Table 5). We present the results from EB smoothed mortality rates by cancer types overall (Supplemental Fig. 1), among NH-white adults (Supplemental Fig. 2), and among NH-black adults (Supplemental Fig. 3). We present the results from LISA analyses by cancer types overall (Supplemental Fig. 4), among NH-white adults (Supplemental Fig. 5), and among NH-black adults (Supplemental Fig. 6). We present the results from LISA analyses by cancer types overall (Supplemental Fig. 7), among NH-white adults (Supplemental Fig. 8), and among NH-black adults (Supplemental Fig. 9).

### Statistical analysis

We present the median and interquartile ranges for county-level community health characteristics due to the non-normal distribution of the continuous variables. We contrasted differences between hot spot and non-hot spot counties for county-level community characteristics using Wilcoxon rank-sum tests. To observe the magnitude of correlation between hot spots and county-level characteristics, we examined the degree of correlation between hot spots and community characteristics using a Spearman correlation (positive values indicate positive correlation) (*ρ*). We used the crude and age-adjusted mortality rates provided by the compressed mortality file (CMF), which uses intercensal (1999, 2001–2009, 2011–2019), and actual (2000 and 2010) U.S. census population estimates. We conducted general linear regression and multivariable (age adjusted) general linear regression modeling with a Poisson distribution to assess the association between hot spot classifications (i.e., hot spot or non-hot spot) and cancer specific mortality rates. We performed statistical analyses using SAS version 9.4. All statistical tests were two-sided and *p*-values ≤ 0.05 were considered statistically significant.

## Results

### Cancer mortality rates by hot spot areas and county-level associated factors

**Figure 1:**
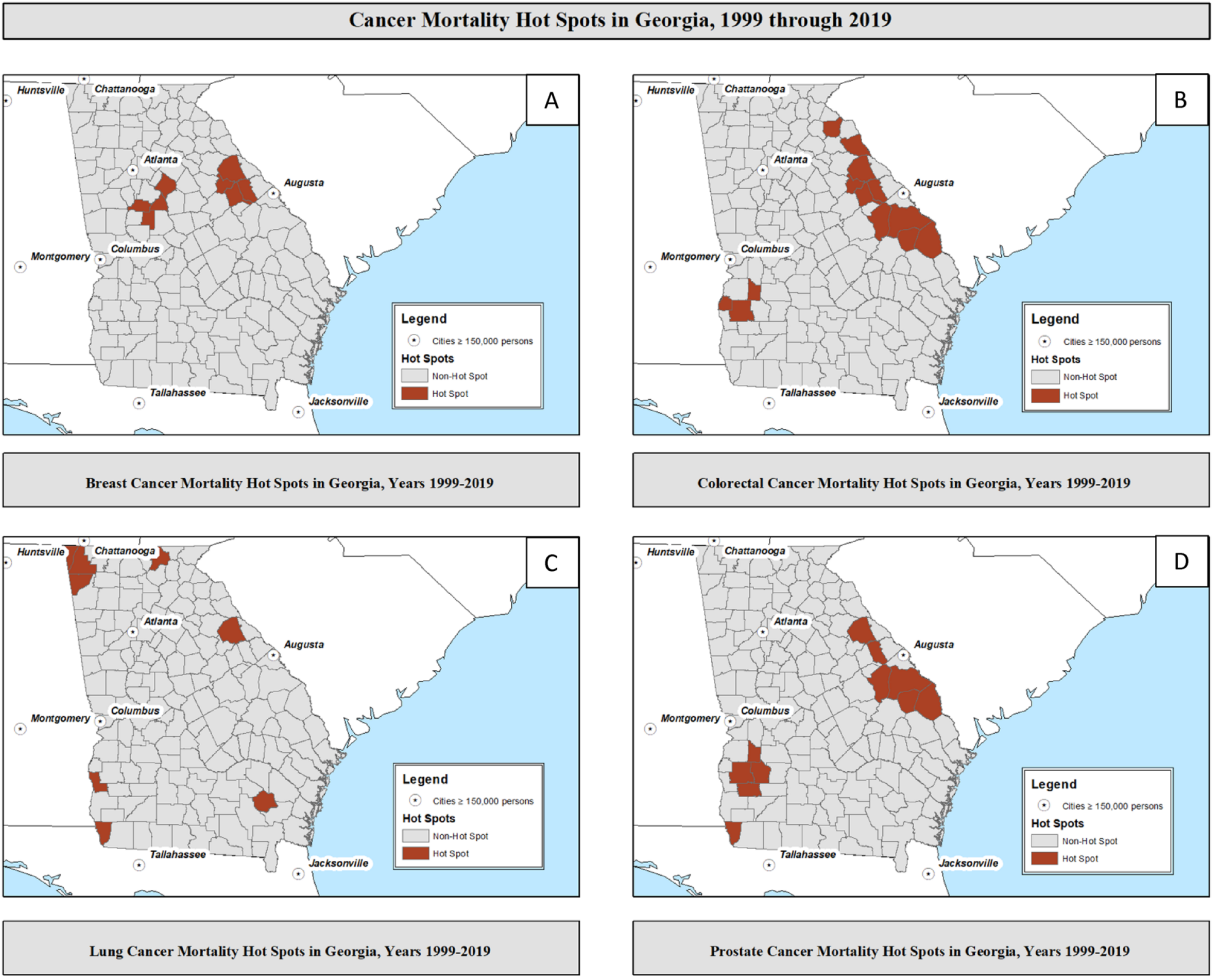
Hot spots of cancer mortality stratified by major cancer types: breast, colorectal, lung, and prostate among all adults in Georgia counties from years 1999 through 2019.

In Georgia, from 1999 and 2019 there were an overall 162,387 cancer deaths observed among adults aged 18 and older; with approximately 24,395 deaths attributed to breast cancer; 29,577 deaths from colorectal cancer, 91,859 deaths from lung cancer, and 16,556 deaths from prostate cancer (Table 1). For all cancers, both crude and age-adjusted mortality rates were higher in the identified hot spot counties when compared to non-hot spot counties. We additionally provided an interactive dashboard for hot spots overall for breast (Supplemental Fig. 10), colorectal (Supplemental Fig. 11), lung (Supplemental Fig. 12), and prostate (Supplemental Fig. 13) [Note: Supplemental Figs. 10–13 are also presented in static form as Fig. 1A–D].

**Table 1:** Patterns of county-level community health risk factors and behaviors by cancer mortality hot spot classification, stratified by cancer types among all adults in Georgia 1999–2019.

Characteristic	Breast cancer		Colorectal cancer		Lung cancer		Prostate cancer	
	Hot spot (*n* = 8)	Non-hot spot (*n* = 151)	Hot spot (*n* = 13)	Non-Hot Spot (*n* = 146)	Hot spot (*n* = 8)	Non-hot spot (*n* = 151)	Hot spot (*n* = 11)	Non-hot spot (*n* = 148)
Cancer-specific deaths ^(1)^	846	23,549	868	28,709	3,076	88,783	453	16,103
Mean annual population ^(2)^	240,129	495,903	189,948	997,765	343,242	962,895	87,536	475,525
Mean crude mortality, 95% CI ^(3)^	49.1 (44.5–54.2)	34.9 (34.0–35.9)	37.5 (34.3–40.9)	24.3 (23.5–25.1)	108.5 (101.5–116.0)	81.9 (80.4–83.3)	49.2 (45.2–53.5)	28.6 (27.7–29.5)
Mean age-adjusted mortality, 95% CI ^(3)^	43.6 (39.0–48.8)	34.4 (33.4–35.4)	31.3 (28.1–35.0)	24.5 (23.7–25.4)	87.7 (81.4–94.4)	79.3 (77.9–80.8)	46.0 (42.0–50.4)	30.4 (29.4–31.3)
	**Presented as Median (Q1–Q3)**							
**Race/ethnicity**								
% NH-white	52.1 (42.0–61.3)	62.1 (52.4–73.2)	50.8 (42.2–53.1)	63.2 (55.4–73.2)	83.2 (56.4–92.1)	61.5 (51.5–71.9)	50.8 (35.6–53.1)	63.4 (55.2–73.6)
% NH-black	41.0 (32.2–49.5)	27.6 (13.8–39.8)	41.9 (40.9–52.4)	27.5 (13.8–36.8)	9.1 (2.8–36.6)	28.0 (16.1–40.4)	41.9 (40.9–60.0)	27.5 (13.0–36.9)
% Hispanic	3.9 (2.8–5.0)	5.1 (3.1–8.2)	3.8 (2.3–4.7)	5.5 (3.3–8.6)	3.5 (2.3–5.2)	5.1 (3.2–8.2)	3.5 (2.7–5.0)	5.5 (3.2–8.4)
% Asian	0.8 (0.6–1.1)	0.9 (0.6–1.6)	0.6 (0.5–0.8)	1.0 (0.7–1.6)	0.7 (0.6–0.8)	1.0 (0.7–1.6)	0.6 (0.5–0.7)	1.0 (0.7–1.6)
**Sex**								
% Female Sex	52.0 (51.2–52.7)	51.0 (50.2–51.9)	52.0 (50.5–52.4)	51.0 (50.2–51.9)	51.2 (50.7–52.1)	51.1 (50.2–52.0)	52.0 (50.1–52.4)	51.0 (50.2–51.9)
**Age**								
% Age < 18 years	21.0 (20.4–24.3)	22.9 (20.3–24.7)	21.1 (20.3–21.7)	23.0 (20.5–24.8)	21.0 (19.8–22.1)	23.0 (20.3–24.8)	21.1 (20.3–23.5)	22.9 (20.4–24.7)
% Age 65 + years	17.7 (16.3–22.9)	17.1 (14.8–19.1)	20.9 (18.3–23.6)	17.0 (14.4–18.8)	21.2 (18.1–25.0)	17.0 (14.6–19.0)	18.8 (16.8–23.1)	17.1 (14.5–19.0)
**Other Characteristics**								
% Some college education	41.6 (36.8–50.7)	50.2 (43.4–59.0)	37.4 (36.2–48.9)	51.0 (43.7–59.3)	47.5 (37.6–51.6)	49.9 (43.1–59.0)	47.1 (36.2–49.0)	50.5 (43.2–59.2)
Median household income	43,139 (38,857–48,577)	43,439 (38,357–52,502)	38,352 (34,090–39,681)	44,498 (38,775–54.016)	41,640 (38,115–45,166)	43,439 (38,357–53,906)	38,187 (33,393–39,361)	44,301 (38,791–53,961)
% Adult smoking	19.2 (18.2–19.5)	18.2 (16.9–19.9)	19.7 (18.4–20.5)	18.1 (16.9–19.7)	18.1 (16.4–19.1)	18.3 (17.0–19.9)	20.5 (19.2–21.4)	18.1 (16.9–19.6)
% Adult obesity	34.7 (31.1–39.0)	34.7 (30.2–37.8)	32.7 (29.0–35.6)	34.7 (30.7–38.2)	33.3 (30.1–36.4)	34.7 (30.3–38.2)	30.0 (27.3–34.5)	34.7 (31.2–38.3)
% Rural population	64.2 (51.2–89.0)	64.7 (40.8–81.3)	75.0 (67.4–88.9)	60.7 (36.8–80.9)	70.3 (62.5–89.7)	61.6 (38.2–81.3)	68.6 (61.0–80.7)	61.4 (37.5–81.6)
% Limited access to healthy foods	5.9 (2.8–11.3)	5.8 (2.5–10.3)	4.8 (2.5–14.3)	5.8 (2.6–10.2)	4.2 (2.4–11.7)	5.9 (2.6–10.3)	4.4 (2.5–9.3)	6.3 (2.6–10.7)
PCP per 10,000 population	4.2 (2.4–4.9)	3.7 (2.2–5.9)	3.2 (1.4–4.0)	3.9 (2.4–6.0)	3.2 (2.2–6.1)	3.8 (2.2–5.6)	3.2 (1.4–4.0)	3.9 (2.4–5.8)

^(1)^Total number of cancer specific deaths from 1999 through 2019. ^(2)^Mean annual population for total period (1999–2019) among counties within hot spot classification. For sex-specific cancers of breast and prostate, these mean population estimates are women and men, respectively. ^(3)^Mortality rates are per 100,000 population. For sex-specific cancers of breast and prostate, they are per 100,000 women and men, respectively. 95% confidence limits presented with rates.

Among all Georgia women, we identified 8 of 159 (5.0%) counties as hot spots for breast cancer mortality, with the majority located within central to east Georgia (Fig. 1A). The hot spots counties (43.6 deaths per 100,000 women, 95% CI: 39.0–48.8) had higher rates than non-hot spots (34.4 deaths per 100,000 women, 95% CI: 33.4–35.4), a higher proportion of NH-black population (41.0% vs. 27.6%, *p* value = 0.01, *ρ* correlation = 0.20), and a lower proportion of adults with some college education (50.2% vs. 41.6%, p value = 0.05, *ρ* correlation = − 0.15) when compared with non-hot spot counties.

We also identified 13 of 159 (8.2%) counties in Georgia as hot spots for colorectal cancer mortality, with one cluster ranging from the north-eastern Piedmont region to eastern Coastal Plains region: and another cluster in southwestern Georgia just south of Columbus (Fig. 1B). Colorectal cancer hot spots had higher proportions of NH-black population (41.9% vs. 27.5%, *p* value < 0.01, *ρ* correlation = 0.27), residents aged 65 and older (20.9% vs. 17.0%, *p* value =  < 0.01, *ρ* correlation = 0.26), lower median household income ($38,352 vs. $44,498, *p* value < 0.01, *ρ* correlation = − 0.26), and rural population (75.0% vs. 60.7%, *p* value = 0.02, *ρ* correlation = 0.19); but lower proportion of population with some college education (37.4% vs. 51.0%, *p* value < 0.01, *ρ* correlation = − 0.25) when compared with non-hot spot counties.

For lung cancer mortality, we identified 8 of 159 (5.0%) counties in Georgia as hot spots, with hot spots identified in four separate quadrants of the state (Fig. 1C). Lung cancer mortality hot spots had higher population aged 65 and older (21.2% vs. 17.0%, *p* value =  < 0.01, *ρ* correlation = 0.22). Though non-significant, lung cancer hot spots had marginally higher proportions of NH-white population (83.2% vs. 61.5%, *p* value = 0.07, *ρ* correlation = 0.15), and rural population (70.3% vs. 61.6%, *p* value = 0.20, *ρ* correlation = 0.10) when compared with non-hot spot counties.

Among all Georgia men, we identified 11 of 159 (6.9%) counties as hot spots for prostate cancer mortality, similarly clustered as colorectal cancer, with one cluster ranging from the north-eastern Piedmont region to eastern Coastal Plains region: and another cluster in southwestern Georgia just outside of Albany, Georgia (Fig. 1D). Prostate cancer hot spot counties had significantly higher proportion of NH-black population (41.9% vs. 27.5%, *p* value < 0.01, *ρ* correlation = 0.31), and lower median household income ($38,187 vs. $44.301, *p* value < 0.01, *ρ* correlation = − 0.27) when compared with non-hot spot counties. Though non-significant, prostate cancer hot spots had more rural population (68.6% vs. 61.4%, *p* value = 0.15, *ρ* correlation = 0.11) when compared with non-hot spot counties.

### Mortality rates by hot spot areas, among non-Hispanic white adults

Among NH-white adults, there were a total of 15,507 deaths attributed to breast cancer, 19,572 deaths attributed to colorectal cancer, 70,938 deaths attributed to lung cancer, and 9,631 deaths attributed to prostate cancer from 1999 through 2019 (Table 2). For all cancers, both crude and age-adjusted mortality rates were higher in the identified NH-white hot spots when compared to non-hot spot counties.

**Table 2:** Patterns of county-level community health risk factors and behaviors by cancer mortality hot spot classification, stratified by cancer types among non-Hispanic white adults in Georgia 1999 – 2019.

Characteristic	Breast cancer		Colorectal cancer		Lung cancer		Prostate cancer	
	Hot spot (*n* = 9)	Non-hot spot (*n* = 150)	Hot spot (*n* = 7)	Non-hot Spot (*n* = 152)	Hot spot (*n* = 7)	Non-hot spot (*n* = 152)	Hot spot (*n* = 8)	Non-hot spot (*n* = 151)
Cancer-specific deaths^(1)^	925	14,582	443	19,129	1,648	69,290	444	9,187
Mean annual population ^(2)^	223,873	291,512	181,850	577,338	186,984	577,102	167,447	277,796
Mean crude mortality, 95% CI ^(3)^	45.2 (41.0–49.8)	36.0 (35.0–37.0)	35.1 (30.9–39.7)	26.1 (25.3–26.9)	141.0 (132.5–150.1)	98.3 (96.7–99.9)	36.9 (32.9–41.4)	25.5 (24.6–26.4)
Mean age-adjusted mortality, 95% CI ^(3)^	44.0 (39.9–48.6)	37.8 (36.7–39.0)	32.4 (28.5–36.9)	26.8 (25.9–27.7)	110.8 (103.3–118.9)	97.4 (95.9–99.0)	33.0 (28.7–37.9)	28.8 (27.8–29.8)
	**Presented as Median (Q1–Q3)**							
**Race/ethnicity**								
% NH-white	58.1 (45.3–66.0)	62.0 (52.0–73.0)	61.4 (50.8–74.6)	62.0 (51.7–72.7)	49.3 (36.2–87.1)	62.2 (52.7–72.7)	64.6 (49.2–82.4)	61.6 (52.0–72.5)
% NH-black	34.1 (28.3–45.5)	27.6 (14.2–39.9)	24.3 (18.9–41.9)	27.9 (14.3–40.0)	47.0 (8.9–60.2)	27.8 (14.5–39.9)	30.0 (2.5–44.4)	27.9 (14.6–40.1)
% Hispanic	4.7 (3.2–5.9)	5.0 (3.1–7.8)	4.6 (3.8–10.2)	5.0 (3.1–7.8)	2.3 (0.2–4.0)	5.4 (3.3–8.2)	4.3 (3.1–6.9)	5.1 (3.1–7.8)
% Asian	1.1 (0.6–1.2)	0.9 (0.7–1.5)	0.8 (0.6–1.2)	0.9 (0.7–1.6)	0.7 (0.3–0.7)	1.0 (0.7–1.6)	0.8 (0.6–1.4)	0.9 (0.7–1.5)
**Sex**								
% Female Sex	52.3 (52.0–53.0)	51.0 (50.2–51.8)	50.5 (50.1–51.0)	51.2 (50.2–52.0)	52.1 (50.5–52.8)	51.1 (50.2–51.9)	52.1 (51.2–52.3)	51.0 (50.2–51.9)
**Age**								
% Age < 18 years	23.5 (20.8–25.1)	22.8 (20.3–24.7)	21.7 (21.0–24.7)	22.8 (20.3–24.7)	20.8 (17.8–21.7)	22.9 (20.4–24.7)	21.6 (18.6–22.8)	23.0 (20.4–24.8)
% Age 65 + years	17.5 (14.4–20.9)	17.1 (14.9–19.1)	18.3 (17.2–19.0)	17.1 (14.7–19.3)	19.0 (18.0–26.8)	17.1 (14.7–19.0)	20.2 (16.2–25.4)	17.1 (14.8–19.0)
**Other characteristics**								
% Some college education	48.9 (45.0–57.0)	49.8 (42.9–59.0)	45.6 (40.3–50.5)	50.1 (43.2–59.0)	51.5 (36.5–56.6)	49.6 (43.2–58.7)	52.8 (46.4–58.4)	49.5 (42.6–59.0)
Median household income	46,059 (43,510–48,831)	42,803 (38,288–51,829)	39,048 (35,841–42,904)	43,723 (38,537–53,204)	34,984 (33,393–42,820)	43,723(38,721–53,204)	42,634 (38,519–45,033)	43,510 (38,357–53,906)
% Adult smoking	18.3 (17.1–18.7)	18.2 (17.0–19.9)	20.2 (18.4–21.7)	18.2 (16.9–19.7)	18.1 (17.4–23.6)	18.3 (16.9–19.8)	18.0 (16.7–20.2)	18.3 (17.1–19.8)
% Adult obesity	36.5 (26.1–39.3)	34.6 (30.3–37.7)	32.8 (29.5–38.8)	34.7 (30.3–38.0)	30.7 (27.3–36.6)	34.7 (30.3–38.2)	31.4 (25.9–34.8)	34.7 (30.3–38.2)
% Rural population	61.0 (31.2–77.9)	65.1 (41.8–81.3)	74.5 (67.0–80.7)	61.1 (39.5–81.6)	73.1 (66.0–100.0)	61.4 (39.5–80.8)	71.8 (62.4–89.6)	61.6 (40.8–81.3)
% Limited access to healthy foods	7.4 (2.8–14.3)	5.8 (2.5–10.2)	2.5 (2.3–6.8)	5.8 (2.6–10.8)	12.0 (5.4–27.7)	5.8 (2.5–10.2)	2.5 (0.8–7.0)	6.1 (2.7–10.7)
PCP per 10,000 population	4.6 (2.5–5.2)	3.7 (2.2–5.6)	3.4 (2.6–3.7)	3.9 (2.2–5.8)	3.3 (1.5–6.8)	3.8 (2.4–5.5)	5.5 (3.5–9.7)	3.7 (2.1–5.4)

^(1)^Total number of cancer specific deaths from 1999 through 2019. ^(2)^Mean annual population for total period (1999–2019) among counties within hot spot classification. For sex-specific cancers of breast and prostate, these mean population estimates are women and men, respectively. ^(3)^Mortality rates are per 100,000 population. For sex-specific cancers of breast and prostate, they are per 100,000 women and men, respectively. 95% confidence limits presented with rates.

### County-level associated factors, among non-Hispanic white adults

**Figure 2:**
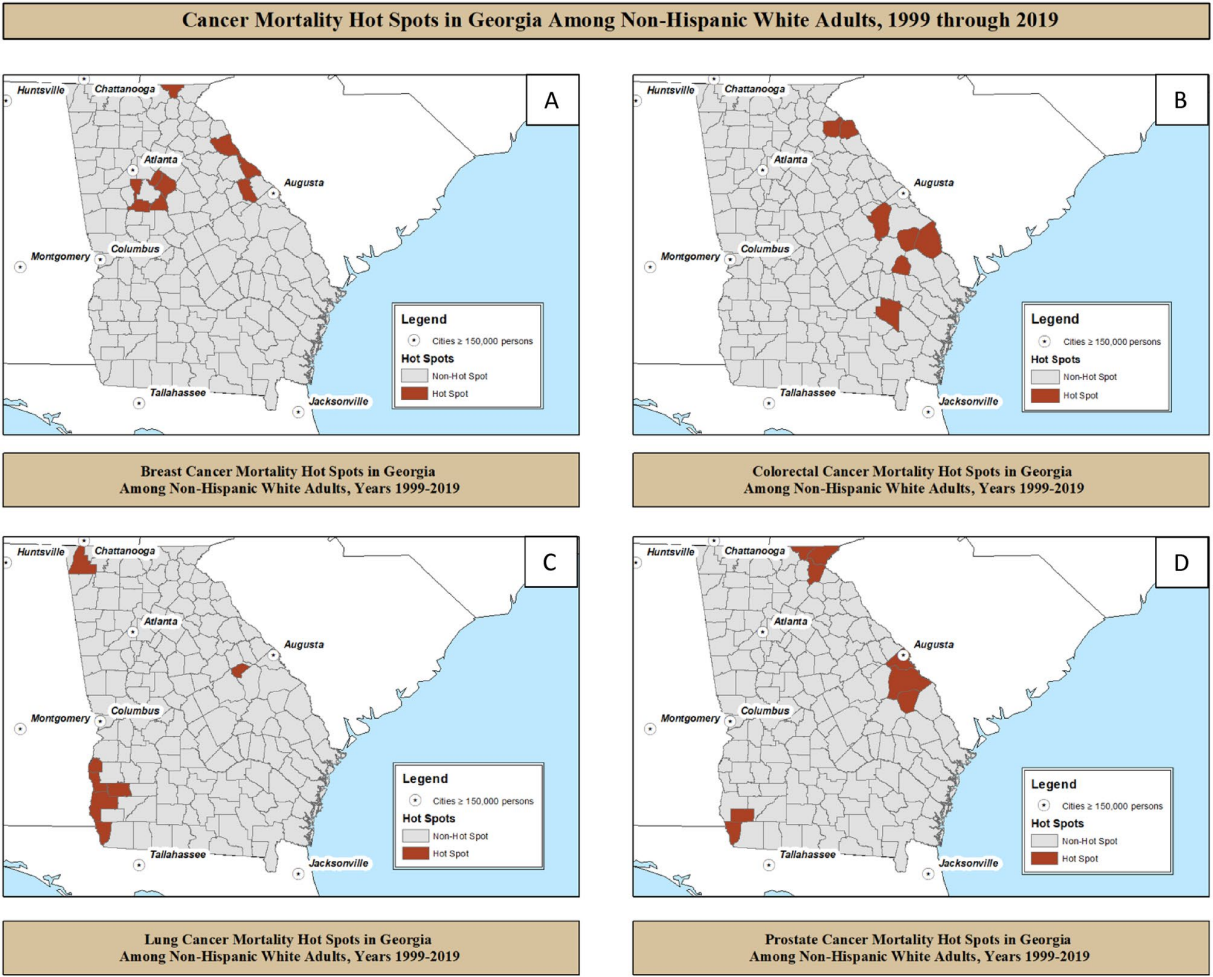
Hot spots of cancer mortality stratified by major cancer types: breast, colorectal, lung, and prostate among non-Hispanic white adults in Georgia counties from years 1999 through 2019.

When stratified by NH-white women, we identified 9 of 159 (5.7%) hot spot counties for breast cancer mortality, with the majority located southwest of Atlanta, eastern Piedmont region, and one county (Towns, Georgia) in Northeastern Blue Ridge region (Fig. 2A). There were no significant differences in county-level factors between NH-white breast cancer hot spots and non-hot spot counties.

Among NH-white adults, we identified 7 of 159 (4.4%) hot spot counties for colorectal cancer mortality, with most hot spots located in the Piedmont region of Eastern Georgia through the Coastal Plain region of southeastern Georgia (Fig. 2B). Colorectal cancer hot spots among NH-white adults had higher proportion of adult smoking (20.2% vs. 18.2%, *p* value = 0.02, *ρ* correlation = 0.19) when compared to non-hot spot counties. Though non-significant, there was marginally higher proportion of rural population (74.5% vs. 61.1%, *p* value = 0.14, *ρ* correlation = 0.12) and lower median household income ($39,048 vs. $43,723, *p* value = 0.11, *ρ* correlation = − 0.13) and in the NH-white colorectal cancer hot spots.

Among NH-white adults, we identified 7 of 159 (4.4%) hot spot counties for lung cancer mortality, with hot spots in three separate quadrants but predominantly in Southwestern Georgia (Fig. 2C). Lung cancer hot spots among NH-white adults had lower median household income ($34,984 vs. $43,723, *p* value < 0.01, *ρ* correlation = − 0.23) when compared to non-hot spot counties. Though non-significant, there was marginally higher proportion of population with limited access to healthy foods (12.0% vs. 5.8%, *p* value = 0.06, *ρ* correlation = 0.15) in NH-white lung cancer hot spots.

For NH-white men, we identified 8 of 159 (5.0%) hot spot counties for prostate cancer mortality, similarly clustered as colorectal cancer, with hot spots in three separate quadrants but predominantly in the Augusta area and Northeastern Blue Ridge region (Fig. 2D). There were no significant differences in county-level factors between NH-white prostate cancer hot spots and non-hot spot counties.

### Mortality rates by hot spot areas, among NH-black adults

Among NH-blacks, there were a total of 7,963 deaths attributed to breast cancer, 8,998 deaths attributed to colorectal cancer, 19,488 deaths attributed to lung cancer, and 9,631 deaths attributed to prostate cancer from 1999 through 2019 (Table 3). For all cancers, both crude and age-adjusted mortality rates were higher in the identified NH-black hot spots when compared to non-hot spot counties.

**Table 3:** Patterns of county-level community health risk factors and behaviors by cancer mortality hot spot classification, stratified by cancer types among NH-black adults in Georgia 1999–2019.

Characteristic	Breast cancer		Colorectal cancer		Lung cancer		Prostate cancer	
	hot spot (*n* = 9)	Non-hot spot (*n* = 150)	Hot spot (*n* = 6)	Non-hot spot (*n* = 153)	Hot spot (*n* = 11)	Non-hot spot (*n* = 148)	Hot spot (*n* = 9)	Non-hot spot (*n* = 150)
Cancer-specific deaths^(1)^	1704	6259	143	8,855	764	18,724	227	6,173
Mean annual population ^(2)^	434,988	131,089	70,761	279,318	85,226	285,288	38,601	128,230
Mean crude mortality, 95% CI ^(3)^	50.6 (46.1–55.4)	39.3 (38.0–40.5)	37.6 (32.6–43.4)	28.2 (27.2–29.2)	83.0 (77.5–88.8)	59.6(58.3–60.9)	68.8 (63.3–74.8)	46.3 (45.0–47.6)
Mean age-adjusted mortality, 95% CI ^(3)^	49.0 (43.7–54.9)	37.4 (35.9–38.9)	27.0 (22.3–32.6)	26.5 (25.4–27.6)	69.2(63.7–75.3)	55.8 (54.4–57.2)	50.2 (44.8–56.2)	44.0 (42.4–45.7)
	**Presented as Median (Q1–Q3)**							
**Race/ethnicity**								
% NH-white	64.6 (57.8–66.0)	61.7 (51.5–73.0)	57.7 (47.5–65.5)	61.8 (52.4–73.0)	53.1 (38.7–70.9)	62.2 (52.7–73.0)	53.1 (50.8–66.5)	62.0 (52.4–73.0)
% NH-black	28.3 (23.7–30.3)	27.8 (13.8–40.4)	34.8 (26.1–46.9)	27.8 (14.4–39.9)	40.8 (14.4–53.5)	27.7 (14.4–39.3)	40.8 (28.2–41.9)	27.6 (14.2–39.8)
% Hispanic	4.0 (3.3–7.3)	5.0 (3.1–8.1)	4.0 (2.3–5.9)	5.1 (3.2–8.1)	4.4 (2.3–5.4)	5.1 (3.2–8.2)	3.3 (2.3–5.3)	5.1 (3.3–8.2)
% Asian	0.7 (0.6–0.8)	1.0 (0.7–1.5)	0.6 (0.5–0.6)	0.9 (0.7–1.6)	0.7 (0.5–1.1)	0.9 (0.7–1.6)	0.6 (0.5–0.7)	1.0 (0.7–1.6)
**Sex**								
% Female Sex	51.6 (51.2–51.8)	51.0 (50.2–52.0)	51.7 (50.5–52.4)	51.1 (50.2–51.9)	52.0 (50.5–52.6)	51.0 (50.2–51.9)	52.0 (51.5–52.4)	51.0 (50.2–51.9)
**Age**								
% Age < 18 years	21.8 (20.6–23.0)	22.9 (20.3–24.8)	20.9 (19.9–21.7)	22.9 (20.4–24.7)	21.5 (20.3–23.2)	22.9 (20.4–24.8)	21.7 (21.1–23.5)	22.9 (20.3–24.7)
% Age 65 + years	18.2 (17.0–20.0)	17.1 (14.8–19.1)	22.3 (18.0–23.9)	17.1 (14.8–19.0)	20.9 (17.6–23.6)	17.0 (14.5–18.9)	18.3 (17.5–20.9)	17.1 (14.6–19.1)
**Other Characteristics**								
% Some college education	50.2 (45.8–59.3)	49.6 (42.6–58.4)	47.8 (37.1–49.0)	50.2 (43.1–59.0)	38.2 (36.2–49.1)	50.5 (43.6–59.0)	48.9 (36.2–53.1)	50.1 (43.1–59.0)
Median household income	51,205 (48,323–57,978)	42,803 (38,352–51,579)	41,233 (38,187–42,820)	43,679 (38,441 52,502)	39,513 (38,187–42,768)	43,990 (38,537–53,961)	40,965 (39,361–42,785)	43,767 (38,357–53,906)
% Adult smoking	17.7 (15.2–19.0)	18.3 (17.1–19.9)	17.6 (17.1–20.5)	18.3 (17.0–19.8)	18.9 (17.4–19.7)	18.2 (16.9–19.9)	19.2 (17.8–20.5)	18.2 (16.9–19.8)
% Adult obesity	34.3 (32.2–34.7)	34.7 (30.2–38.2)	34.4 (25.8–36.1)	34.7 (30.3–38.1)	32.5 (25.8–37.7)	34.7 (30.5–38.2)	28.7 (25.6–34.5)	34.7 (31.0–38.2)
% Rural population	75.4 (60.9–80.9)	63.1 (41.6–81.3)	78.0 (70.6–100.0)	61.6 (40.8–80.7)	67.4 (50.6–100.0)	63.5 (39.5–80.8)	70.6 (66.1–80.7)	61.4 (38.2–81.3)
% Limited access to healthy foods	6.7 (3.0–8.1)	5.8 (2.5–10.7)	9.6 (1.3–15.6)	5.8 (2.6–10.2)	11.5 (3.1–15.6)	5.8 (2.5–10.0)	4.8 (2.8–14.3)	5.8 (2.6–10.2)
PCP per 10,000 population	4.3 (3.6–5.4)	3.7 (2.2–5.6)	3.4 (2.8–3.7)	3.9 (2.2–5.7)	4.0 (0.8–5.7)	3.7 (2.3–5.5)	3.6 (3.3–5.1)	3.7 (2.1–5.9)

^(1)^Total number of cancer specific deaths from 1999 through 2019. ^(2)^Mean annual population for total period (1999–2019) among counties within hot spot classification. For sex-specific cancers of breast and prostate, these mean population estimates are women and men, respectively. ^(3)^Mortality rates are per 100,000 population. For sex-specific cancers of breast and prostate, they are per 100,000 women and men, respectively. 95% confidence limits presented with rates.

### County-level associated factors, among NH-black adults

**Figure 3:**
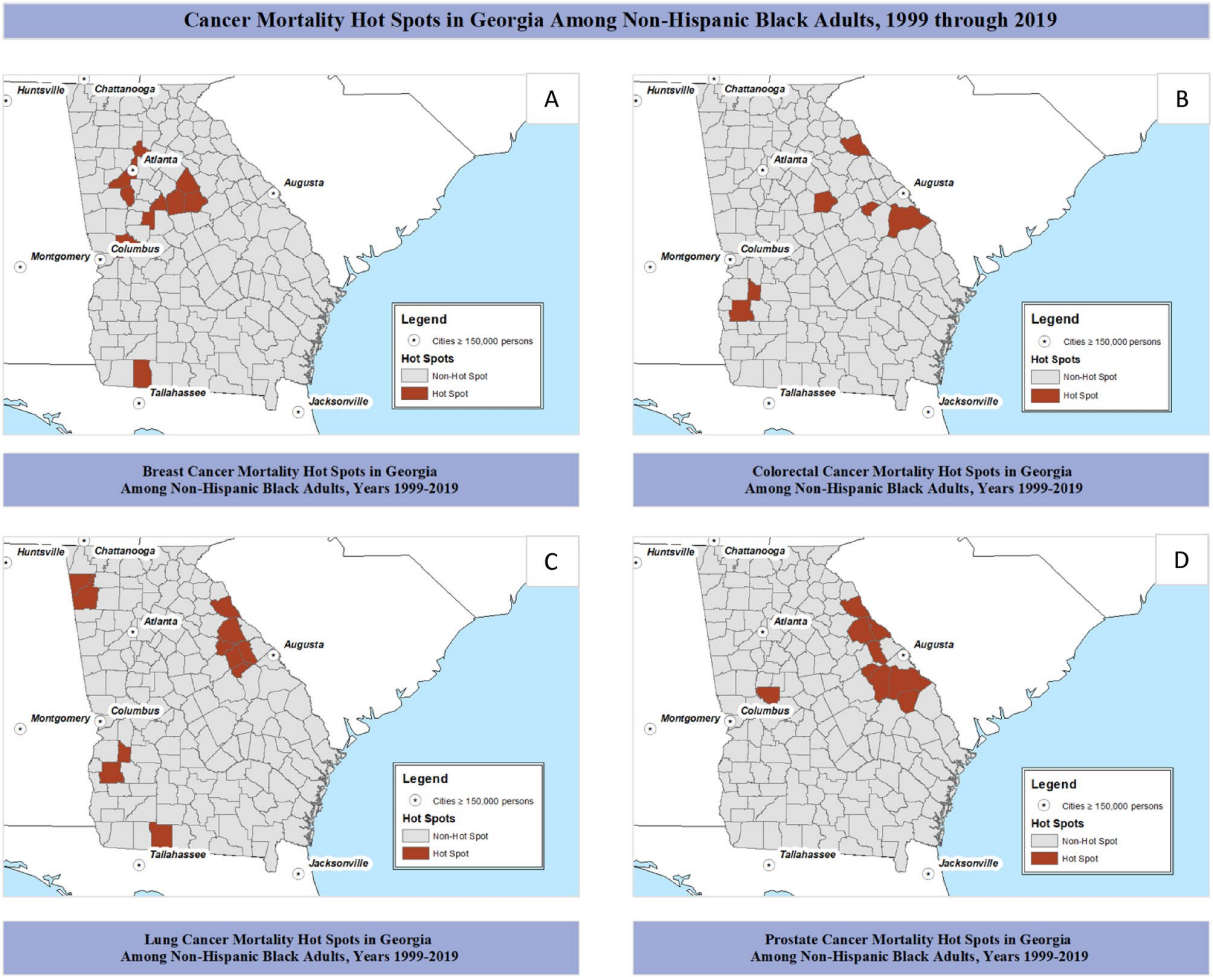
Hot spots of cancer mortality stratified by major cancer types: breast, colorectal, lung, and prostate among NH-black adults in Georgia counties from years 1999 through 2019.

When stratified by NH-black women, we identified 9 of 159 (5.7%) hot spot counties for breast cancer mortality, with the majority in metro-Atlanta and 40 miles east of Atlanta (Fig. 3A). NH-black breast cancer hot spots had lower median household income ($51,205 vs. $42,803, *p* value = 0.15, *ρ* correlation = − 0.08) when compared with non-hot spots. There were no significant differences in county-level factors between NH-black breast cancer hot spots and non-hot spot counties.

Among NH-black adults, we identified 6 of 159 (3.8%) hot spot counties for colorectal cancer mortality, with most hot spots located in the Piedmont region in eastern Georgia or within southwestern Georgia (Fig. 3B). NH-black colorectal cancer hot spots had a higher proportion of population aged 65 and older (22.3% vs. 17.1%, *p* value = 0.03, *ρ* correlation = 0.17) when compared to non-hot spot counties. Though non-significant, there was marginally lower proportion of college educated population (47.8% vs. 50.2%, *p* value = 0.25, *ρ* correlation = − 0.09) and the median household income was ($43,679 vs. $41.233, *p* value = 0.29, *ρ* correlation = − 0.08) lower in NH-black colorectal cancer hot spots compared with non-hot spots among NH-black adults.

Among NH-black adults, we identified 11 of 159 (6.9%) hot spot counties for lung cancer mortality, with hot spots in three separate quadrants including the eastern Piedmont region, southwestern Georgia, and the Ridge and Valley region located in northwestern Georgia (Fig. 3C). Lung cancer hot spots among NH-black adults had higher prevalence of population aged 65 and older (20.9% vs. 17.0%, *p* value < 0.01, *ρ* correlation = 0.23); and though non-significant, a higher proportion of population with limited access to healthy foods (11.5% vs. 5.8%, *p* value = 0.09, *ρ* correlation = 0.13) when compared to non-hot spots. NH-black lung cancer hot spots had significantly less population with some college education (38.2% vs. 50.5%, *p* value = 0.03, *ρ* correlation = − 0.18) when compared to non-hot spots.

When stratified by NH-black men, we identified 9 of 159 (5.7%) hot spot counties for prostate cancer mortality, with hot spots predominantly in the Augusta area and eastern Piedmont to Coastal Plain regions (Fig. 3D). NH-black hot spots were characterized by marginally higher proportion of population with lower proportion of adult obesity (28.7% vs. 34.7%, *p* value = 0.02, *ρ* correlation = − 0.18); and though non-significant, there was a higher proportion of NH-black residents (40.8% vs. 27.6, *p* value = 0.12, *ρ* correlation = 0.12) and greater proportion of rural residents (70.6% vs. 61.4%, *p* value = 0.14, *ρ* correlation = 0.12).

## Discussion

The results of this study indicate that there are striking geographic patterns in breast, colorectal, lung, and prostate cancer mortality in Georgia, and that elevations in cancer mortality are correlated with county-level characteristics including higher NH-black population, older adult population, greater poverty, and rurality. Overall, the age-adjusted mortality rates for all cancers were higher in hot spots when compared to non-hot spot counties, ranging from as low as 11% greater in lung cancer hot spots to nearly 50% higher in prostate cancer hot spots. Among NH-white adults, the age-adjusted rates for all cancers were higher in hot spots compared to non-hot spot counties, ranging from 14% higher in lung cancer hot spots to 21% higher in the colorectal cancer hot spots. Generally, among NH-white adults, hot spots had higher NH-black population, greater poverty, rurality, and limited access to healthy foods, compared with non-hot spots. Among NH-black adults, the age-adjusted rates for all cancers were higher in hot spots compared to non-hot spots, ranging from 2% higher in colorectal cancer hot spots to 31% higher in the breast cancer hot spots. Among NH-black adults, in general, hot spots compared with non-hot spots had higher NH-black population, greater poverty, and rurality. Lastly, we observed distinct geographic patterns in hot spots for each cancer overall, and by race. Of great interest, many geographic clusters of hot spots were in the (1) eastern Piedmont to Coastal Plain regions, (2) southwestern rural Georgia area, and (3) northernmost rural Georgia areas. These findings are novel, given that our study is the first to identify hot spots of all four major cancers in Georgia and examine the county-level determinants of cancer outcomes specific to hot spot designations.

NH-black adults disproportionately suffer poorer cancer morbidity and mortality when compared with NH-white adults; as evidenced by NH-black adults in the U.S. having only 63% overall 5-year relative survival for all cancers when compared to 68% among NH-white adults (2). In our study, age-adjusted mortality rates among NH-black adults hot spots for both breast (49.0 vs. 44.0, per 100,000 population) and prostate (50.2 vs. 33.0, per 100,000 population) cancers where higher than NH-white hot spots for breast and prostate cancers. Given that early detection through cancer screening impacts morbidity, mortality, and cancer survival, this factor may have substantial relevancy. In a recent study among NH-black men treated at an academic medical center within east Georgia, Coughlin et al.^34^ observed that only 38% of men had received a prostate-specific antigen (PSA) test within the past year and the men’s knowledge of prostate cancer was only fair to good^34^. It is possible that within these identified hot spots among NH-black adults, barriers such as inadequate access to and availability of health care services, lack of knowledge of cancer prevention and screening recommendations, culturally inappropriate or insensitive cancer control programming, low health literacy, and medical distrust, all mediate the association between race and cancer mortality^35–37^.

Those living in rural communities often experience many barriers to appropriate care across the cancer continuum including inadequate screening, follow-up of abnormal cancer screening tests, travel time and distance from cancer care providers, and treatment of diagnosed cancers^38^. We observed that regardless of race/ethnicity, hot spots were frequently characterized by greater rural population, higher poverty (lower household income), and a greater population with limited education. It is plausible that those residing in hot spots are less likely to get appropriate screening, which in turn increases risk of later stage disease and lower cancer survival. Social determinants of health including socioeconomic status, neighborhood disadvantage, unemployment, racial discrimination, social support, medical distrust, housing insecurity, and geographic factors are associated with cancer outcomes^11,39^. Marginalized and under-resourced communities (i.e., rural, lower education, underinsured) have poorer access to screening resources including mammography^40^, low-dose computed tomography (LDCT)^41,42^ and/or lower likelihood of receiving colorectal cancer screening and prostate cancer screening within past year^43^. Tailor et al.^42^ found that rural populations and specifically census tracts with greater uninsured and under-educated (less than high school education) populations have greater distances to computerized tomography facilities^42^. The incidence of cancers associated with modifiable risk factors like tobacco use and receipt of screening modalities are generally higher in rural communities^44^. Atkins et al.^45^ reported that lung cancer mortality increased in a dose-dependent function with increasing stage of rural–urban continuum area (RUCA) code, as those living in the most rural US communities had a 91% higher risk of lung cancer mortality compared to those living in the most urban communities^45^.

Rural populations experience 3% higher cancer incidence and 10% higher cancer mortality when compared to urban populations^46,47^. Further, Zahnd et al.^44^ explained that rural populations had lower incidence of localized stage cancers and higher incidence of distant stage cancers when compared to urban populations^14^. Future research is needed to examine the differences in rural–urban patient cancer survivorship and determine the etiologic mechanisms (e.g., social-behavioral, clinical characteristics, environmental) that explain the disparities observed in rural communities. Studies that examine geographic patterns in cancer incidence and treatment are also needed.

Obesity is a significant risk for cancer and is associated with 13 cancers, constituting more than 40% of all diagnosed cancers. We observed that for the overall population, there were no significant differences in obesity prevalence between hot spot counties and non-hot spot counties. Not finding correlations between hot spots and obesity may be due to misclassification of obesity status or to the homogeneity of obesity across all Georgia populations (i.e., on average 33% of all Georgia counties have adult obesity). We observed that several hot spot counties were associated with higher proportion of older adult population; those aged 65 and older were more likely to live in several hot spots. Zeng et al.^48^ reported that there is a widening gap in cancer survival between younger and older patients and suggest that this may be explained by differential utilization of newer treatment among the older adult population with the older adult population less likely to engage in novel treatment modalities ^48^.

### Limitations

There are a few strengths and limitations of concern when making interpretations of these results. First, the CDC NCHS suppresses data for counties that observed less than 10 deaths over the observation period. Further, very rural, and small counties may not have been identified as hot spots because the deaths are not reported, or the causes of death are unknown. This in turn could reflect an underestimation of the overall mortality rates that we present. Furthermore, it is plausible that counties identified as hot spots near borders of Georgia have limited information to borrow for geospatial measures and we may have observed a statistical edge effect. Nevertheless, for all analyses focused on breast cancer mortality, the major hot spot identified was located within northcentral Georgia (outside of Atlanta, Georgia) for all race/ethnicities and when stratified among NH-white and NH-black adults. Furthermore, one of the key strengths from the Bayesian approach is that for counties with very limited data, it allowed us to borrow strength from the observed data to derive a reasonable posterior estimation for cancer mortality rates. We were unable to discern specific cancer characteristics as CDC mortality data only allows for identification of death attributed to specific cancers. Because we utilized the CDC’s underlying causes of death file, it is plausible that we underestimated the true burden of cancer mortality. However, this study aimed to focus on cancer-related deaths and limiting the analyses to cancer disease or events leading directly to deaths allows for more conservative estimation of hot spots. In addition, this study was ecologic, and thus the results are not able to discern causality but rather patterns and associations at an aggregate level (county-level). We are unable to deduce causality but rather associations on the larger county-level scale. To date, there have been limited studies to examine the geographic distribution of cancer subtypes, clinical and molecular phenotypes, and treatment (i.e., radiation, surgery, chemotherapy). Future studies should examine hot spots in Georgia based on incidence and patient data through SEER, further elucidate the relationship between our identified cancer-specific hot spots with cancer-specific survival, and the mediating role of clinicodemographic factors could be useful in mitigating cancer health inequities.

## Conclusion

Georgia is a state affected by syndemic problems including cancer, poverty, and racial and geographic disparities. Nearly 54% (85/159) of the counties in Georgia are classified as rural based on 2013 Rural–Urban Continuum Codes (RUCC). Moreover, rural communities are disproportionately burdened by persistent poverty and higher neighborhood disadvantages (i.e., limited employment, education, income)^49,50^. Further, Georgia ranks 14^th^ in overall incident cancer cases with nearly 60,000 new cases of cancer in 2022 alone (1). To mitigate continued disparities observed in cancer outcomes for Georgia, it is important that we focus on modifiable risk factors through clinical, policy and population-based interventions, as well as to understand the unique barriers experienced by rural and underserved populations. It is critically important to simultaneously collaborate with these communities when implementing culturally tailored programs and interventions aimed at reducing cancer morbidity and mortality over the next few decades. We additionally created an interactive dashboard with estimates of cancer hot spots via an online database, which may allow for researchers, community stakeholders, and policymakers to examine and utilize these granular and specific disparities when deciding resource allocation and identifying programs and interventions that aim to reduce the burden of excess cancer morbidity and mortality in disparate communities. Concerted cancer prevention and control efforts including transportation, increased availability of affordable screening, and quality treatment facilities should provide great utility in reducing cancer burden in the observed cancer hot spot areas. Importantly, future work should aim to provide more culturally tailored approaches for racial/ethnic populations and rural communities along the cancer care continuum and be a cooperative effort between community organizations, local health centers (i.e., Federally Qualified Health Centers), and larger Cancer Centers (i.e., Georgia Cancer Center).

## Supplementary Information

Supplementary Information 1.

Supplementary Information 2.

Supplementary Information 3.

Supplementary Information 4.

Supplementary Information 5.

Supplementary Information 6.

Supplementary Information 7.

Supplementary Information 8.

Supplementary Information 9.

Supplementary Information 10.

Supplementary Information 11.

Supplementary Information 12.

Supplementary Information 13.

Supplementary Information 14.

Supplementary Information 15.

Supplementary Information 16.

Supplementary Information 17.

Supplementary Information 18.

Supplementary Information 19.
