# Supplementary figures and images for: Understanding geographic and racial/ethnic disparities in mortality from four major cancers in the state of Georgia: a spatial epidemiologic analysis, 1999–2019
Source: Sci Rep. 2022 Aug 19;12:14143. doi: 10.1038/s41598-022-18374-7 (PMC9391349; doi:10.1038/s41598-022-18374-7)

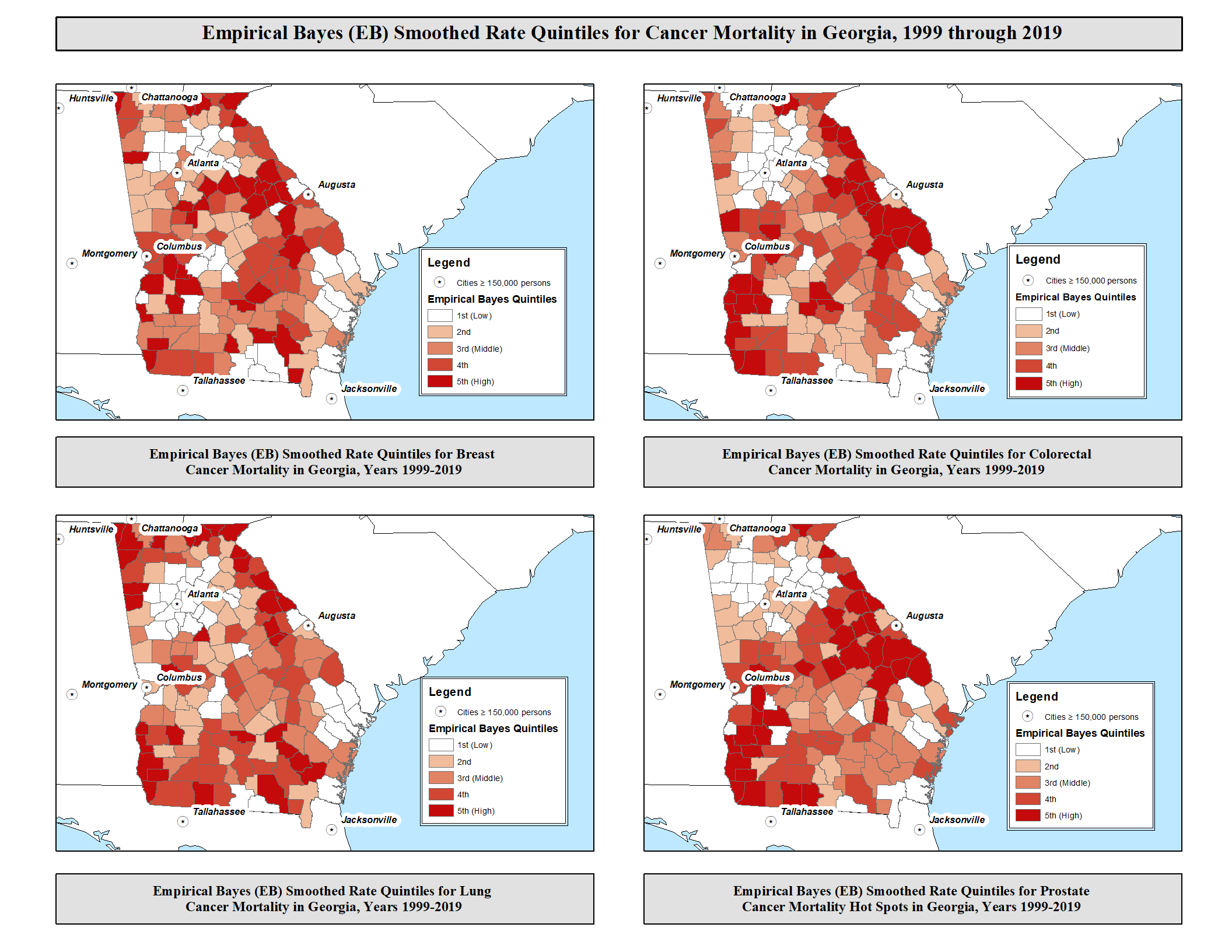

Supplement: Supplementary file 1 — Supplementary Information 1. [file 41598_2022_18374_MOESM1_ESM.tif]

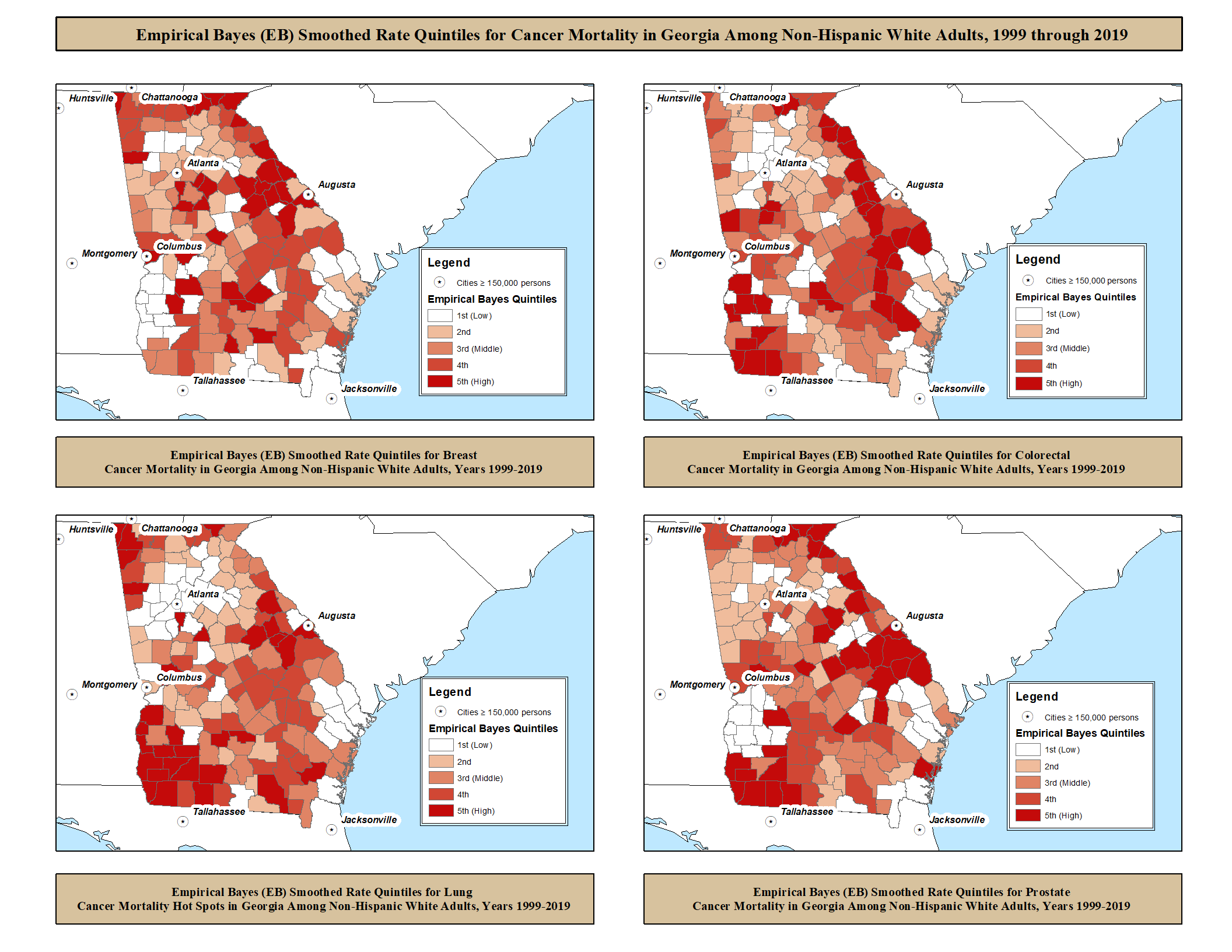

Supplement: Supplementary file 2 — Supplementary Information 2. [file 41598_2022_18374_MOESM2_ESM.tif]

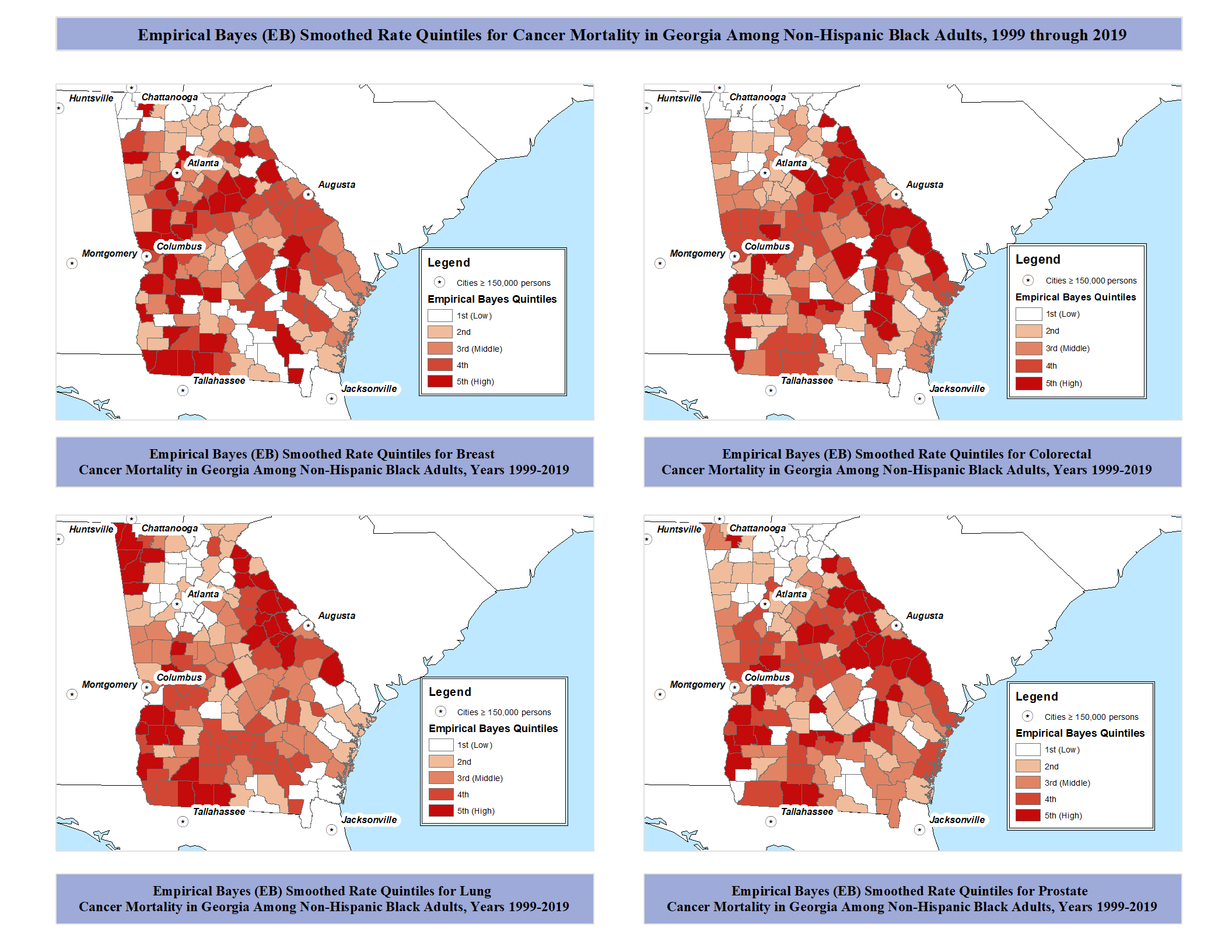

Supplement: Supplementary file 3 — Supplementary Information 3. [file 41598_2022_18374_MOESM3_ESM.tif]

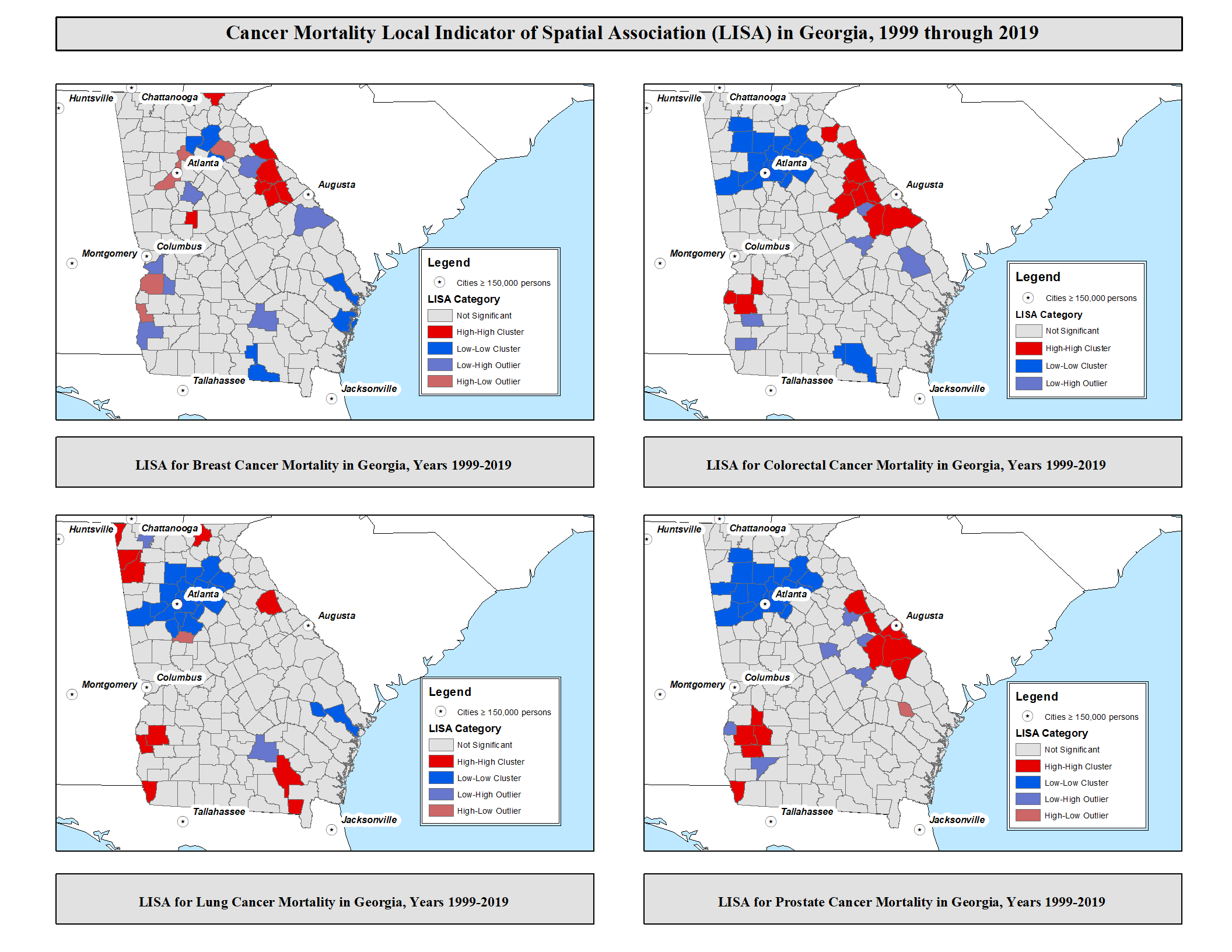

Supplement: Supplementary file 4 — Supplementary Information 4. [file 41598_2022_18374_MOESM4_ESM.tif]

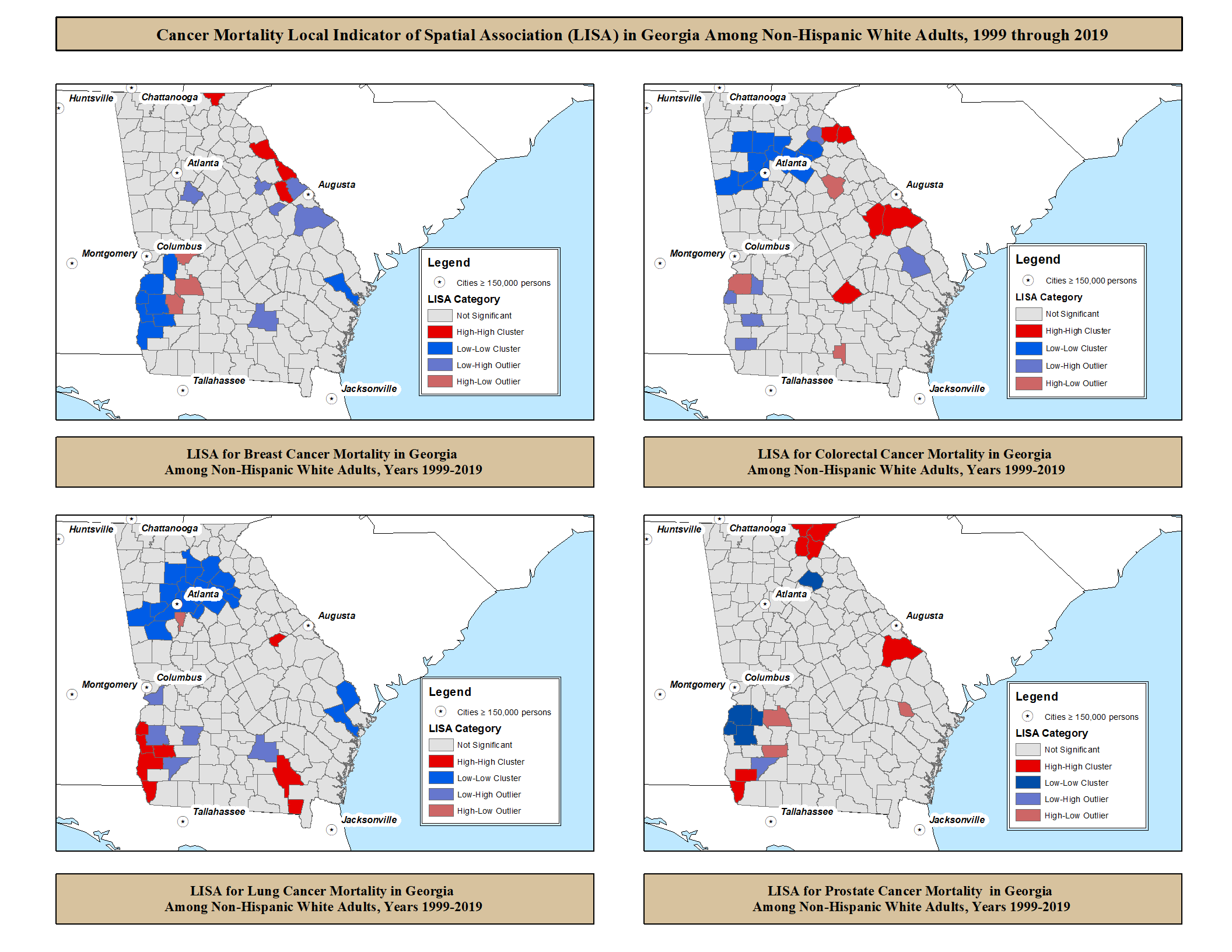

Supplement: Supplementary file 5 — Supplementary Information 5. [file 41598_2022_18374_MOESM5_ESM.tif]

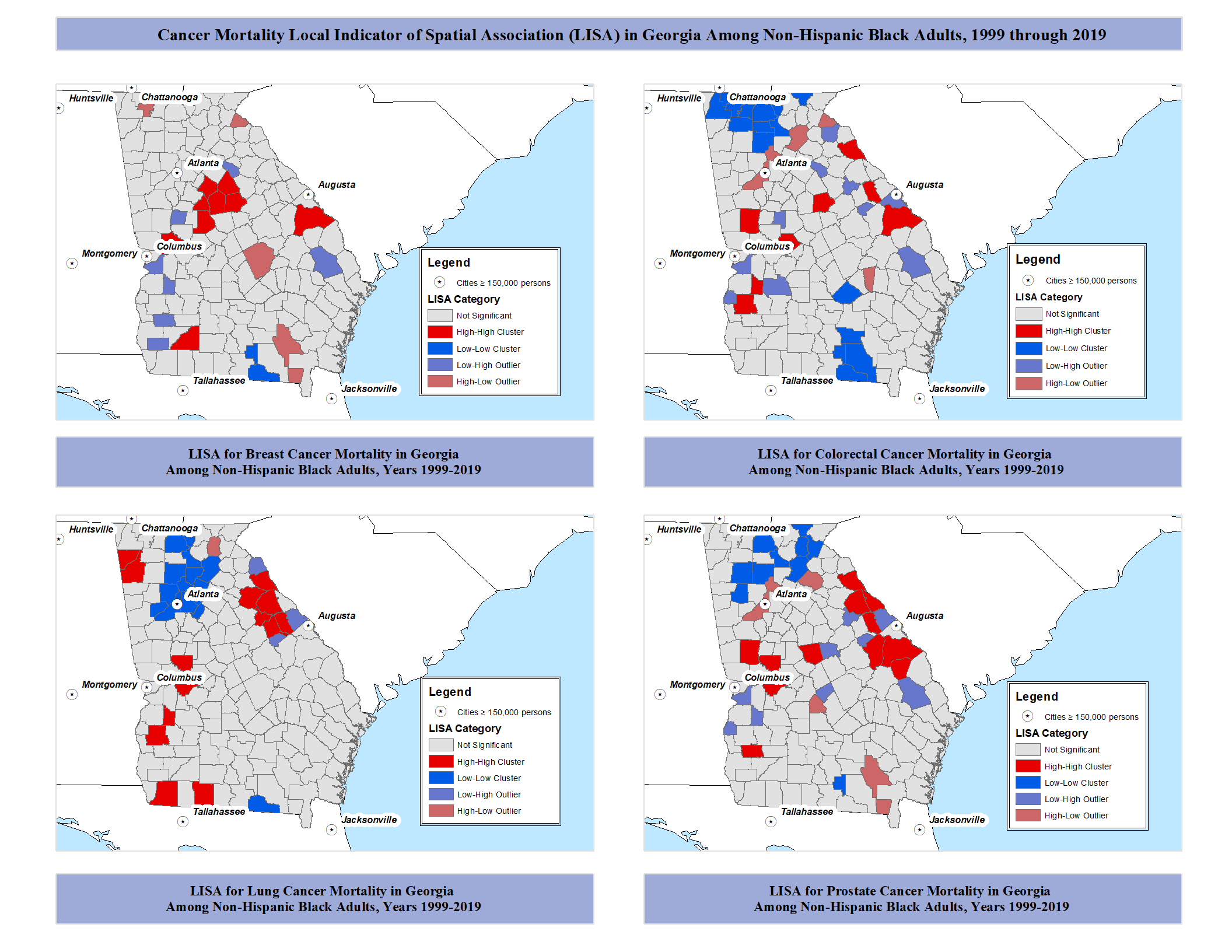

Supplement: Supplementary file 6 — Supplementary Information 6. [file 41598_2022_18374_MOESM6_ESM.tif]

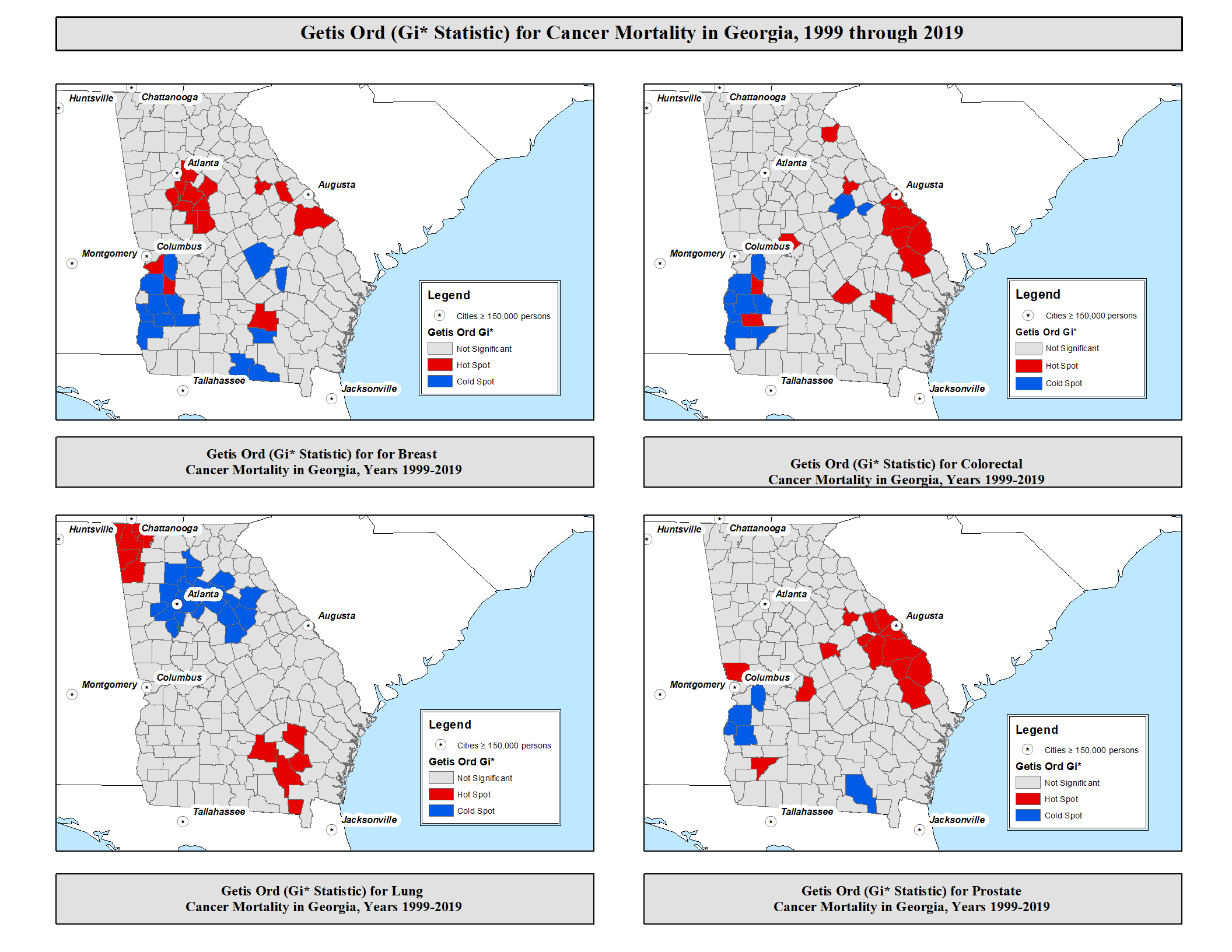

Supplement: Supplementary file 7 — Supplementary Information 7. [file 41598_2022_18374_MOESM7_ESM.tif]

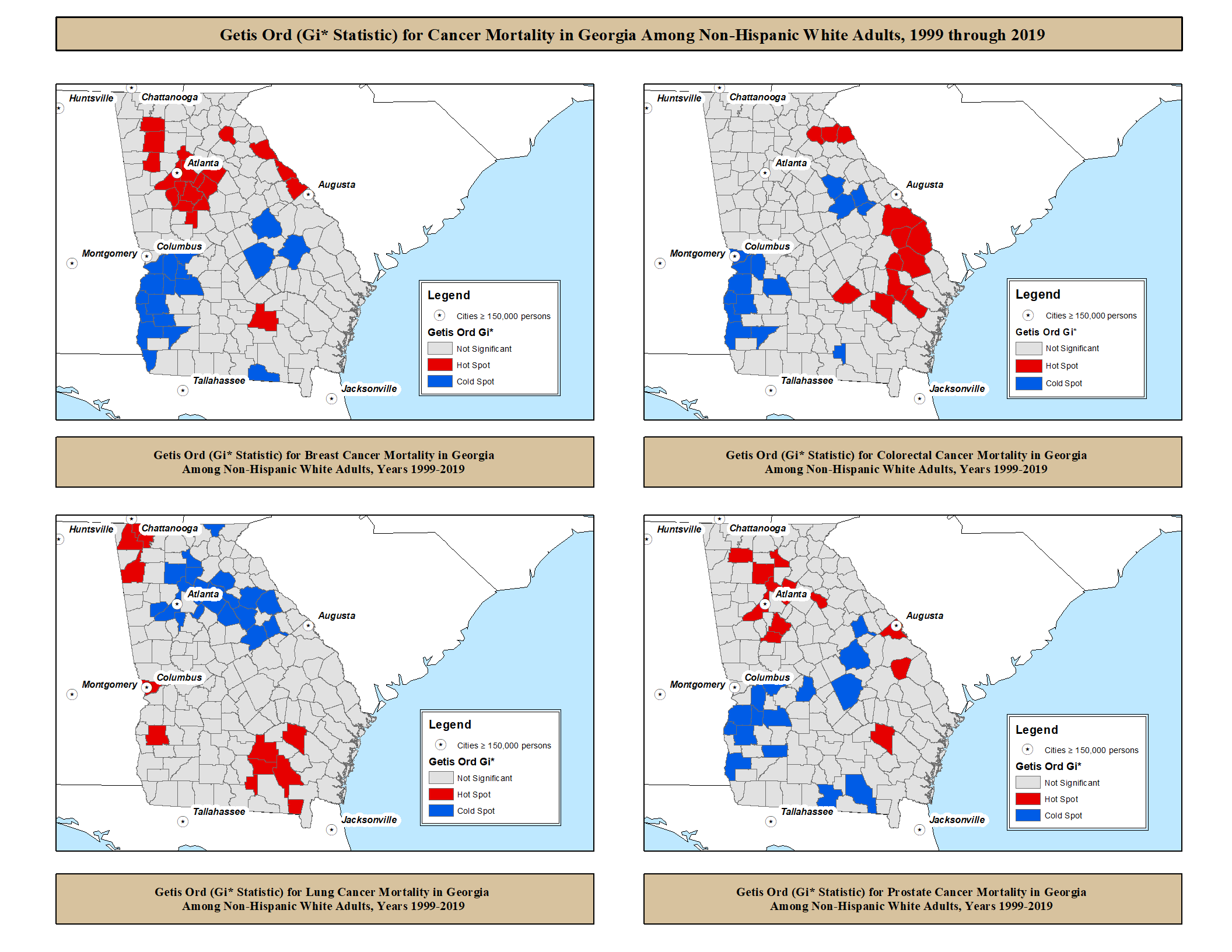

Supplement: Supplementary file 8 — Supplementary Information 8. [file 41598_2022_18374_MOESM8_ESM.tif]

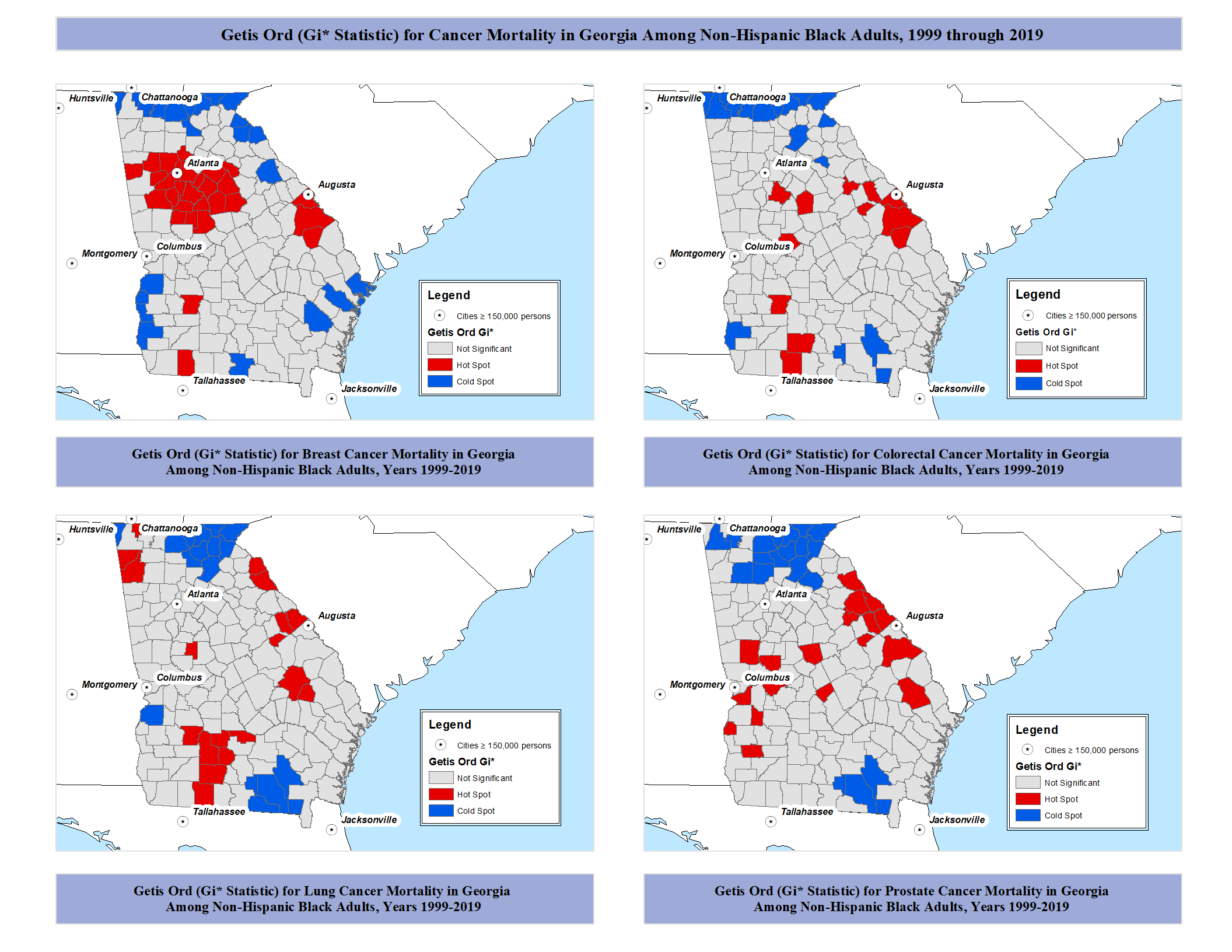

Supplement: Supplementary file 9 — Supplementary Information 9. [file 41598_2022_18374_MOESM9_ESM.tif]
