# Supplementary material for: Understanding geographic and racial/ethnic disparities in mortality from four major cancers in the state of Georgia: a spatial epidemiologic analysis, 1999–2019
Source: Sci Rep. 2022 Aug 19;12:14143. doi: 10.1038/s41598-022-18374-7 (PMC9391349; doi:10.1038/s41598-022-18374-7)
Supplement: Supplementary file 11 — Supplementary Information 11. [file 41598_2022_18374_MOESM11_ESM.docx]

**Supplemental Figure 11**. **Interactive dashboard for Georgia colorectal cancer hot spots:** [**https://www.arcgis.com/apps/dashboards/116526a127aa4550b0daf9f5dea1ec77**](https://www.arcgis.com/apps/dashboards/116526a127aa4550b0daf9f5dea1ec77)

NOTE: This figure is presented as Figure 1B “Colorectal Cancer Mortality Hot Spots in Georgia, Years 1999 - 2019” in the main manuscript file. We have provided this interactive dashboard for readership to have more accessibility to the overall colorectal cancer hot spots.
