# Supplementary material for: Understanding geographic and racial/ethnic disparities in mortality from four major cancers in the state of Georgia: a spatial epidemiologic analysis, 1999–2019
Source: Sci Rep. 2022 Aug 19;12:14143. doi: 10.1038/s41598-022-18374-7 (PMC9391349; doi:10.1038/s41598-022-18374-7)
Supplement: Supplementary file 14 — Supplementary Information 14. [file 41598_2022_18374_MOESM14_ESM.docx]

**Supplemental Material:**

**Figures**

**Supplemental Figure 1:** Empirical Bayes (EB) smoothed rate quintiles for cancer mortality in Georgia, 1999 through 2019. Map was created using ArcGIS 10.7 (1)

**Supplemental Figure 2:** Empirical Bayes (EB) smoothed rate quintiles for cancer mortality in Georgia among non-Hispanic white adults, 1999 through 2019. Map was created using ArcGIS 10.7 (1)

**Supplemental Figure 3:** Empirical Bayes (EB) smoothed rate quintiles for cancer mortality in Georgia among non-Hispanic black adults, 1999 through 2019. Map was created using ArcGIS 10.7 (1)

**Supplemental Figure 4:** Cancer mortality Local Indicator of Spatial Association (LISA) in Georgia, 1999 through 2019. Map was created using ArcGIS 10.7 (1)

**Supplemental Figure 5:** Cancer mortality Local Indicator of Spatial Association (LISA) in Georgia among non-Hispanic white adults, 1999 through 2019. Map was created using ArcGIS 10.7 (1)

**Supplemental Figure 6:** Cancer mortality Local Indicator of Spatial Association (LISA) in Georgia among non-Hispanic black adults, 1999 through 2019. Map was created using ArcGIS 10.7 (1)

**Supplemental Figure 7:** Getis Ord (Gi* statistic) for cancer mortality in Georgia, 1999 through 2019. Map was created using ArcGIS 10.7 (1)

**Supplemental Figure 8:** Getis Ord (Gi* statistic) for cancer mortality in Georgia among non-Hispanic white adults, 1999 through 2019. Map was created using ArcGIS 10.7 (1)

**Supplemental Figure 9:** Getis Ord (Gi* statistic) for cancer mortality in Georgia among non-Hispanic black adults, 1999 through 2019. Map was created using ArcGIS 10.7 (1)

**Supplemental Figure 10. Interactive dashboard for Georgia breast cancer hot spots:** [**https://www.arcgis.com/apps/dashboards/079353fba9864dc0917e0aef2925c602**](https://www.arcgis.com/apps/dashboards/079353fba9864dc0917e0aef2925c602)

**Supplemental Figure 11**. **Interactive dashboard for Georgia colorectal cancer hot spots:** [**https://www.arcgis.com/apps/dashboards/116526a127aa4550b0daf9f5dea1ec77**](https://www.arcgis.com/apps/dashboards/116526a127aa4550b0daf9f5dea1ec77)

**Supplemental Figure 12. Interactive dashboard for Georgia lung cancer hot spots:** [**https://www.arcgis.com/apps/dashboards/58036d32fc3744d896e47e61676b0af1**](https://www.arcgis.com/apps/dashboards/58036d32fc3744d896e47e61676b0af1)

**Supplemental Figure 13. Interactive dashboard for Georgia prostate cancer hot spots:** [**https://www.arcgis.com/apps/dashboards/b6b28a6658444f7ba2d88c7d5eef1e9f**](https://www.arcgis.com/apps/dashboards/b6b28a6658444f7ba2d88c7d5eef1e9f)

**Tables**

**Supplemental Table 1.** Detailed definitions and technical information for 2020 County Health Rankings (CHR) community health characteristics used in study analysis.

**Supplemental Table 2.** List of counties for Georgia breast cancer mortality hot spots, 1999 through 2019.

**Supplemental Table 3.** List of counties for Georgia colorectal cancer mortality hot spots, 1999 through 2019.

**Supplemental Table 4.** List of counties for Georgia lung cancer mortality hot spots, 1999 through 2019.

**Supplemental Table 5.** List of counties for Georgia prostate cancer mortality hot spots, 1999 through 2019.

**Materials**

**Supplemental Material 1.** Detailed description of geospatial methodology.

REFERENCES

1. ESRI. 2019 ArcMap 10.7. Environmental Systems Research Institute <<https://desktop.arcgis.com/en/arcmap/10.7/get-started/terms-of-use/copyright-information.htm>>. Accessed 2022.
