# Supplementary material for: Understanding geographic and racial/ethnic disparities in mortality from four major cancers in the state of Georgia: a spatial epidemiologic analysis, 1999–2019
Source: Sci Rep. 2022 Aug 19;12:14143. doi: 10.1038/s41598-022-18374-7 (PMC9391349; doi:10.1038/s41598-022-18374-7)
Supplement: Supplementary file 15 — Supplementary Information 15. [file 41598_2022_18374_MOESM15_ESM.docx]

| **Supplemental Table 1: Detailed definitions and technical information for 2020 County Health Rankings (CHR) community characteristics used in study analysis. All variables are county-level proportions.** | |
| --- | --- |
|  |  |
| **Community Characteristic** | **Definition and/or Technical Information** |
|  |  |
| Non-Hispanic White population | The proportion of the population that are non-Hispanic White. Data are originally from 2018 US Census population estimates via 2020 CHR. |
| Non-Hispanic Black or African American population | The proportion of the population that are African American. Data are originally from 2018 US Census population estimates via 2020 CHR. |
| Hispanic population | The proportion of the population that are Hispanic. Hispanic population consisted of those that identified as having Hispanic, Latino, or Spanish origins including but not limited to; Mexican American, Chicano, Puerto Rican, Cuban, Argentinean, Colombian, Dominican, Nicaraguan, Salvadoran, and Spaniard Data are originally from 2018 US Census population estimates via 2020 CHR. |
| Asian population | The proportion of the population that are Asian. Data are originally from 2018 US Census population estimates via 2020 CHR. |
| Below 18 years of age | The proportion of the population that are under 18 years. Data are originally from 2018 US Census population estimates via 2020 CHR. |
| 65 years of age and older | The proportion of the population that are 65 years of age and older. Data are originally from 2018 US Census population estimates via 2020 CHR. |
| Adult obesity | The proportion of the adult population (age 20 and older) that reports a body mass index (BMI) greater than or equal to 30 kg/m^2^. |
| Adult smoking | The proportion of the adult population that currently smokes every day or most days and has smoked at least 100 cigarettes in their lifetime. |
| Some college education | Proportion of adult population with some college education, those that attende. Data are originally taken from the 2018 American Community Survey via the 2020 CHR. The 5-Year Summary File provides estimates of average characteristics from 2014 through 2018. |
| Median household income | Median household income. Data are originally taken from Small Area Income and Poverty Estimates via the 2020 CHR. |
| Limited access to healthy foods | The proportion of the population that is low income and does not live close to a grocery store. Living close to a grocery store is defined differently in rural and non-rural areas; in rural areas, it means living less than ten miles from a grocery store; in non-rural areas, less than one mile. "Low income" is defined as having an annual family income of less than or equal to 200 percent of the federal poverty threshold for the family size. Data are from 2015 United States Department of Agriculture (USDA) via 2020 CHR. |
| PCP per 10,000 persons | The ratio of the population to total primary care physicians. Primary care physicians include non-federal, practicing physicians (M.D.'s and D.O.'s) under age 75 specializing in general practice medicine, family medicine, internal medicine, and pediatrics. Data are from 2017 Area Health Resource File/ American Medical Association via 2020 CHR. |
