# Supplementary material for: Understanding geographic and racial/ethnic disparities in mortality from four major cancers in the state of Georgia: a spatial epidemiologic analysis, 1999–2019
Source: Sci Rep. 2022 Aug 19;12:14143. doi: 10.1038/s41598-022-18374-7 (PMC9391349; doi:10.1038/s41598-022-18374-7)
Supplement: Supplementary file 16 — Supplementary Information 16. [file 41598_2022_18374_MOESM16_ESM.docx]

| **Obs** | **County** | **GEOID** | **Breast Cancer Mortality Hot Spots for All Women** | **Breast Cancer Mortality Hot Spots for African American Women** | **Breast Cancer Mortality Hot Spots for NH-White Women** | **Empirical Bayes Smoothed Mortality Rate, All Women per 100,000** |
| --- | --- | --- | --- | --- | --- | --- |
| **1** | Appling County, GA | 13001 | Non-Hot Spot | Non-Hot Spot | Non-Hot Spot | 35.5167 |
| **2** | Atkinson County, GA | 13003 | Non-Hot Spot | Non-Hot Spot | Non-Hot Spot | 40.1827 |
| **3** | Bacon County, GA | 13005 | Non-Hot Spot | Non-Hot Spot | Non-Hot Spot | 36.3238 |
| **4** | Baker County, GA | 13007 | Non-Hot Spot | Non-Hot Spot | Non-Hot Spot | 34.7038 |
| **5** | Baldwin County, GA | 13009 | Non-Hot Spot | Non-Hot Spot | Non-Hot Spot | 31.7697 |
| **6** | Banks County, GA | 13011 | Non-Hot Spot | Non-Hot Spot | Non-Hot Spot | 29.9241 |
| **7** | Barrow County, GA | 13013 | Non-Hot Spot | Non-Hot Spot | Non-Hot Spot | 27.9215 |
| **8** | Bartow County, GA | 13015 | Non-Hot Spot | Non-Hot Spot | Non-Hot Spot | 29.4044 |
| **9** | Ben Hill County, GA | 13017 | Non-Hot Spot | Non-Hot Spot | Non-Hot Spot | 45.8619 |
| **10** | Berrien County, GA | 13019 | Non-Hot Spot | Non-Hot Spot | Non-Hot Spot | 32.9575 |
| **11** | Bibb County, GA | 13021 | Non-Hot Spot | Non-Hot Spot | Non-Hot Spot | 37.0928 |
| **12** | Bleckley County, GA | 13023 | Non-Hot Spot | Non-Hot Spot | Non-Hot Spot | 31.6897 |
| **13** | Brantley County, GA | 13025 | Non-Hot Spot | Non-Hot Spot | Non-Hot Spot | 31.2199 |
| **14** | Brooks County, GA | 13027 | Non-Hot Spot | Non-Hot Spot | Non-Hot Spot | 34.1636 |
| **15** | Bryan County, GA | 13029 | Non-Hot Spot | Non-Hot Spot | Non-Hot Spot | 31.8979 |
| **16** | Bulloch County, GA | 13031 | Non-Hot Spot | Non-Hot Spot | Non-Hot Spot | 28.4698 |
| **17** | Burke County, GA | 13033 | Non-Hot Spot | Non-Hot Spot | Non-Hot Spot | 34.5716 |
| **18** | Butts County, GA | 13035 | Hot Spot | Hot Spot | Hot Spot | 49.4084 |
| **19** | Calhoun County, GA | 13037 | Non-Hot Spot | Non-Hot Spot | Non-Hot Spot | 35.4108 |
| **20** | Camden County, GA | 13039 | Non-Hot Spot | Non-Hot Spot | Non-Hot Spot | 29.4250 |
| **21** | Candler County, GA | 13043 | Non-Hot Spot | Non-Hot Spot | Non-Hot Spot | 33.5106 |
| **22** | Carroll County, GA | 13045 | Non-Hot Spot | Non-Hot Spot | Non-Hot Spot | 32.2662 |
| **23** | Catoosa County, GA | 13047 | Non-Hot Spot | Non-Hot Spot | Non-Hot Spot | 31.6234 |
| **24** | Charlton County, GA | 13049 | Non-Hot Spot | Non-Hot Spot | Non-Hot Spot | 30.7180 |
| **25** | Chatham County, GA | 13051 | Non-Hot Spot | Non-Hot Spot | Non-Hot Spot | 31.2011 |
| **26** | Chattahoochee County, | 13053 | Non-Hot Spot | Non-Hot Spot | Non-Hot Spot | 35.9932 |
| **27** | Chattooga County, GA | 13055 | Non-Hot Spot | Non-Hot Spot | Non-Hot Spot | 37.2092 |
| **28** | Cherokee County, GA | 13057 | Non-Hot Spot | Non-Hot Spot | Non-Hot Spot | 26.6577 |
| **29** | Clarke County, GA | 13059 | Non-Hot Spot | Non-Hot Spot | Non-Hot Spot | 23.5950 |
| **30** | Clay County, GA | 13061 | Non-Hot Spot | Non-Hot Spot | Non-Hot Spot | 43.7816 |
| **31** | Clayton County, GA | 13063 | Non-Hot Spot | Non-Hot Spot | Hot Spot | 28.8584 |
| **32** | Clinch County, GA | 13065 | Non-Hot Spot | Non-Hot Spot | Non-Hot Spot | 28.3100 |
| **33** | Cobb County, GA | 13067 | Non-Hot Spot | Non-Hot Spot | Non-Hot Spot | 28.3545 |
| **34** | Coffee County, GA | 13069 | Non-Hot Spot | Non-Hot Spot | Non-Hot Spot | 34.4148 |
| **35** | Colquitt County, GA | 13071 | Non-Hot Spot | Non-Hot Spot | Non-Hot Spot | 34.1976 |
| **36** | Columbia County, GA | 13073 | Non-Hot Spot | Non-Hot Spot | Non-Hot Spot | 30.6208 |
| **37** | Cook County, GA | 13075 | Non-Hot Spot | Non-Hot Spot | Non-Hot Spot | 43.1033 |
| **38** | Coweta County, GA | 13077 | Non-Hot Spot | Non-Hot Spot | Non-Hot Spot | 31.4204 |
| **39** | Crawford County, GA | 13079 | Non-Hot Spot | Non-Hot Spot | Non-Hot Spot | 34.0249 |
| **40** | Crisp County, GA | 13081 | Non-Hot Spot | Non-Hot Spot | Non-Hot Spot | 35.3262 |
| **41** | Dade County, GA | 13083 | Non-Hot Spot | Non-Hot Spot | Non-Hot Spot | 40.1589 |
| **42** | Dawson County, GA | 13085 | Non-Hot Spot | Non-Hot Spot | Non-Hot Spot | 28.6567 |
| **43** | Decatur County, GA | 13087 | Non-Hot Spot | Non-Hot Spot | Non-Hot Spot | 37.0594 |
| **44** | DeKalb County, GA | 13089 | Non-Hot Spot | Non-Hot Spot | Non-Hot Spot | 30.8422 |
| **45** | Dodge County, GA | 13091 | Non-Hot Spot | Non-Hot Spot | Non-Hot Spot | 36.5489 |
| **46** | Dooly County, GA | 13093 | Non-Hot Spot | Non-Hot Spot | Non-Hot Spot | 32.9364 |
| **47** | Dougherty County, GA | 13095 | Non-Hot Spot | Non-Hot Spot | Non-Hot Spot | 33.8315 |
| **48** | Douglas County, GA | 13097 | Non-Hot Spot | Non-Hot Spot | Non-Hot Spot | 32.2486 |
| **49** | Early County, GA | 13099 | Non-Hot Spot | Non-Hot Spot | Non-Hot Spot | 33.0937 |
| **50** | Echols County, GA | 13101 | Non-Hot Spot | Non-Hot Spot | Non-Hot Spot | 20.5063 |
| **51** | Effingham County, GA | 13103 | Non-Hot Spot | Non-Hot Spot | Non-Hot Spot | 29.7750 |
| **52** | Elbert County, GA | 13105 | Non-Hot Spot | Non-Hot Spot | Hot Spot | 37.8291 |
| **53** | Emanuel County, GA | 13107 | Non-Hot Spot | Non-Hot Spot | Non-Hot Spot | 39.6122 |
| **54** | Evans County, GA | 13109 | Non-Hot Spot | Non-Hot Spot | Non-Hot Spot | 30.2562 |
| **55** | Fannin County, GA | 13111 | Non-Hot Spot | Non-Hot Spot | Non-Hot Spot | 42.5795 |
| **56** | Fayette County, GA | 13113 | Non-Hot Spot | Hot Spot | Non-Hot Spot | 34.3962 |
| **57** | Floyd County, GA | 13115 | Non-Hot Spot | Non-Hot Spot | Non-Hot Spot | 34.5757 |
| **58** | Forsyth County, GA | 13117 | Non-Hot Spot | Non-Hot Spot | Non-Hot Spot | 20.9435 |
| **59** | Franklin County, GA | 13119 | Non-Hot Spot | Non-Hot Spot | Non-Hot Spot | 35.8089 |
| **60** | Fulton County, GA | 13121 | Non-Hot Spot | Hot Spot | Non-Hot Spot | 33.9504 |
| **61** | Gilmer County, GA | 13123 | Non-Hot Spot | Non-Hot Spot | Non-Hot Spot | 34.3231 |
| **62** | Glascock County, GA | 13125 | Non-Hot Spot | Non-Hot Spot | Non-Hot Spot | 26.4590 |
| **63** | Glynn County, GA | 13127 | Non-Hot Spot | Non-Hot Spot | Non-Hot Spot | 33.1334 |
| **64** | Gordon County, GA | 13129 | Non-Hot Spot | Non-Hot Spot | Non-Hot Spot | 31.5659 |
| **65** | Grady County, GA | 13131 | Non-Hot Spot | Hot Spot | Non-Hot Spot | 37.3080 |
| **66** | Greene County, GA | 13133 | Non-Hot Spot | Non-Hot Spot | Non-Hot Spot | 47.7873 |
| **67** | Gwinnett County, GA | 13135 | Non-Hot Spot | Non-Hot Spot | Non-Hot Spot | 24.0298 |
| **68** | Habersham County, GA | 13137 | Non-Hot Spot | Non-Hot Spot | Non-Hot Spot | 35.9920 |
| **69** | Hall County, GA | 13139 | Non-Hot Spot | Non-Hot Spot | Non-Hot Spot | 27.0412 |
| **70** | Hancock County, GA | 13141 | Non-Hot Spot | Non-Hot Spot | Non-Hot Spot | 43.4491 |
| **71** | Haralson County, GA | 13143 | Non-Hot Spot | Non-Hot Spot | Non-Hot Spot | 32.0709 |
| **72** | Harris County, GA | 13145 | Non-Hot Spot | Non-Hot Spot | Non-Hot Spot | 37.2904 |
| **73** | Hart County, GA | 13147 | Non-Hot Spot | Non-Hot Spot | Non-Hot Spot | 35.9797 |
| **74** | Heard County, GA | 13149 | Non-Hot Spot | Non-Hot Spot | Non-Hot Spot | 31.3822 |
| **75** | Henry County, GA | 13151 | Non-Hot Spot | Non-Hot Spot | Non-Hot Spot | 32.0665 |
| **76** | Houston County, GA | 13153 | Non-Hot Spot | Non-Hot Spot | Non-Hot Spot | 29.0237 |
| **77** | Irwin County, GA | 13155 | Non-Hot Spot | Non-Hot Spot | Non-Hot Spot | 35.0263 |
| **78** | Jackson County, GA | 13157 | Non-Hot Spot | Non-Hot Spot | Non-Hot Spot | 30.6847 |
| **79** | Jasper County, GA | 13159 | Non-Hot Spot | Hot Spot | Non-Hot Spot | 38.3194 |
| **80** | Jeff Davis County, GA | 13161 | Non-Hot Spot | Non-Hot Spot | Non-Hot Spot | 35.4848 |
| **81** | Jefferson County, GA | 13163 | Non-Hot Spot | Non-Hot Spot | Non-Hot Spot | 40.6771 |
| **82** | Jenkins County, GA | 13165 | Non-Hot Spot | Non-Hot Spot | Non-Hot Spot | 38.0661 |
| **83** | Johnson County, GA | 13167 | Non-Hot Spot | Non-Hot Spot | Non-Hot Spot | 37.6252 |
| **84** | Jones County, GA | 13169 | Non-Hot Spot | Non-Hot Spot | Non-Hot Spot | 32.4442 |
| **85** | Lamar County, GA | 13171 | Hot Spot | Hot Spot | Non-Hot Spot | 38.4865 |
| **86** | Lanier County, GA | 13173 | Non-Hot Spot | Non-Hot Spot | Non-Hot Spot | 24.3101 |
| **87** | Laurens County, GA | 13175 | Non-Hot Spot | Non-Hot Spot | Non-Hot Spot | 36.0193 |
| **88** | Lee County, GA | 13177 | Non-Hot Spot | Non-Hot Spot | Non-Hot Spot | 28.3787 |
| **89** | Liberty County, GA | 13179 | Non-Hot Spot | Non-Hot Spot | Non-Hot Spot | 19.1720 |
| **90** | Lincoln County, GA | 13181 | Non-Hot Spot | Non-Hot Spot | Hot Spot | 37.6223 |
| **91** | Long County, GA | 13183 | Non-Hot Spot | Non-Hot Spot | Non-Hot Spot | 22.4939 |
| **92** | Lowndes County, GA | 13185 | Non-Hot Spot | Non-Hot Spot | Non-Hot Spot | 22.6707 |
| **93** | Lumpkin County, GA | 13187 | Non-Hot Spot | Non-Hot Spot | Non-Hot Spot | 30.7436 |
| **94** | McDuffie County, GA | 13189 | Hot Spot | Non-Hot Spot | Hot Spot | 38.5385 |
| **95** | McIntosh County, GA | 13191 | Non-Hot Spot | Non-Hot Spot | Non-Hot Spot | 30.2201 |
| **96** | Macon County, GA | 13193 | Non-Hot Spot | Non-Hot Spot | Non-Hot Spot | 30.1216 |
| **97** | Madison County, GA | 13195 | Non-Hot Spot | Non-Hot Spot | Non-Hot Spot | 34.9897 |
| **98** | Marion County, GA | 13197 | Non-Hot Spot | Non-Hot Spot | Non-Hot Spot | 41.5672 |
| **99** | Meriwether County, GA | 13199 | Non-Hot Spot | Non-Hot Spot | Non-Hot Spot | 34.2350 |
| **100** | Miller County, GA | 13201 | Non-Hot Spot | Non-Hot Spot | Non-Hot Spot | 38.5891 |
| **101** | Mitchell County, GA | 13205 | Non-Hot Spot | Non-Hot Spot | Non-Hot Spot | 35.0684 |
| **102** | Monroe County, GA | 13207 | Non-Hot Spot | Non-Hot Spot | Non-Hot Spot | 33.0035 |
| **103** | Montgomery County, GA | 13209 | Non-Hot Spot | Non-Hot Spot | Non-Hot Spot | 35.9768 |
| **104** | Morgan County, GA | 13211 | Non-Hot Spot | Hot Spot | Non-Hot Spot | 38.9894 |
| **105** | Murray County, GA | 13213 | Non-Hot Spot | Non-Hot Spot | Non-Hot Spot | 33.6979 |
| **106** | Muscogee County, GA | 13215 | Non-Hot Spot | Non-Hot Spot | Non-Hot Spot | 38.3369 |
| **107** | Newton County, GA | 13217 | Hot Spot | Non-Hot Spot | Hot Spot | 39.4006 |
| **108** | Oconee County, GA | 13219 | Non-Hot Spot | Non-Hot Spot | Non-Hot Spot | 28.9903 |
| **109** | Oglethorpe County, GA | 13221 | Non-Hot Spot | Non-Hot Spot | Non-Hot Spot | 32.2483 |
| **110** | Paulding County, GA | 13223 | Non-Hot Spot | Non-Hot Spot | Non-Hot Spot | 28.2336 |
| **111** | Peach County, GA | 13225 | Non-Hot Spot | Non-Hot Spot | Non-Hot Spot | 28.9578 |
| **112** | Pickens County, GA | 13227 | Non-Hot Spot | Non-Hot Spot | Non-Hot Spot | 33.2288 |
| **113** | Pierce County, GA | 13229 | Non-Hot Spot | Non-Hot Spot | Non-Hot Spot | 36.4054 |
| **114** | Pike County, GA | 13231 | Non-Hot Spot | Non-Hot Spot | Non-Hot Spot | 36.8321 |
| **115** | Polk County, GA | 13233 | Non-Hot Spot | Non-Hot Spot | Non-Hot Spot | 40.4842 |
| **116** | Pulaski County, GA | 13235 | Non-Hot Spot | Non-Hot Spot | Non-Hot Spot | 29.7392 |
| **117** | Putnam County, GA | 13237 | Non-Hot Spot | Hot Spot | Non-Hot Spot | 33.6301 |
| **118** | Quitman County, GA | 13239 | Non-Hot Spot | Non-Hot Spot | Non-Hot Spot | 14.6318 |
| **119** | Rabun County, GA | 13241 | Non-Hot Spot | Non-Hot Spot | Non-Hot Spot | 38.8557 |
| **120** | Randolph County, GA | 13243 | Non-Hot Spot | Non-Hot Spot | Non-Hot Spot | 31.2132 |
| **121** | Richmond County, GA | 13245 | Non-Hot Spot | Non-Hot Spot | Non-Hot Spot | 36.8554 |
| **122** | Rockdale County, GA | 13247 | Non-Hot Spot | Non-Hot Spot | Hot Spot | 34.0122 |
| **123** | Schley County, GA | 13249 | Non-Hot Spot | Non-Hot Spot | Non-Hot Spot | 40.4674 |
| **124** | Screven County, GA | 13251 | Non-Hot Spot | Non-Hot Spot | Non-Hot Spot | 36.1710 |
| **125** | Seminole County, GA | 13253 | Non-Hot Spot | Non-Hot Spot | Non-Hot Spot | 40.8628 |
| **126** | Spalding County, GA | 13255 | Hot Spot | Non-Hot Spot | Hot Spot | 43.3904 |
| **127** | Stephens County, GA | 13257 | Non-Hot Spot | Non-Hot Spot | Non-Hot Spot | 42.5115 |
| **128** | Stewart County, GA | 13259 | Non-Hot Spot | Non-Hot Spot | Non-Hot Spot | 48.0764 |
| **129** | Sumter County, GA | 13261 | Non-Hot Spot | Non-Hot Spot | Non-Hot Spot | 42.5172 |
| **130** | Talbot County, GA | 13263 | Non-Hot Spot | Hot Spot | Non-Hot Spot | 37.9214 |
| **131** | Taliaferro County, GA | 13265 | Hot Spot | Non-Hot Spot | Non-Hot Spot | 51.6903 |
| **132** | Tattnall County, GA | 13267 | Non-Hot Spot | Non-Hot Spot | Non-Hot Spot | 34.6496 |
| **133** | Taylor County, GA | 13269 | Non-Hot Spot | Non-Hot Spot | Non-Hot Spot | 36.3735 |
| **134** | Telfair County, GA | 13271 | Non-Hot Spot | Non-Hot Spot | Non-Hot Spot | 44.5867 |
| **135** | Terrell County, GA | 13273 | Non-Hot Spot | Non-Hot Spot | Non-Hot Spot | 40.8200 |
| **136** | Thomas County, GA | 13275 | Non-Hot Spot | Non-Hot Spot | Non-Hot Spot | 37.0443 |
| **137** | Tift County, GA | 13277 | Non-Hot Spot | Non-Hot Spot | Non-Hot Spot | 34.7678 |
| **138** | Toombs County, GA | 13279 | Non-Hot Spot | Non-Hot Spot | Non-Hot Spot | 37.3957 |
| **139** | Towns County, GA | 13281 | Non-Hot Spot | Non-Hot Spot | Hot Spot | 38.2433 |
| **140** | Treutlen County, GA | 13283 | Non-Hot Spot | Non-Hot Spot | Non-Hot Spot | 37.6153 |
| **141** | Troup County, GA | 13285 | Non-Hot Spot | Non-Hot Spot | Non-Hot Spot | 32.2341 |
| **142** | Turner County, GA | 13287 | Non-Hot Spot | Non-Hot Spot | Non-Hot Spot | 35.6355 |
| **143** | Twiggs County, GA | 13289 | Non-Hot Spot | Non-Hot Spot | Non-Hot Spot | 31.5771 |
| **144** | Union County, GA | 13291 | Non-Hot Spot | Non-Hot Spot | Non-Hot Spot | 46.5522 |
| **145** | Upson County, GA | 13293 | Non-Hot Spot | Non-Hot Spot | Non-Hot Spot | 35.3033 |
| **146** | Walker County, GA | 13295 | Non-Hot Spot | Non-Hot Spot | Non-Hot Spot | 37.5439 |
| **147** | Walton County, GA | 13297 | Non-Hot Spot | Non-Hot Spot | Non-Hot Spot | 31.9658 |
| **148** | Ware County, GA | 13299 | Non-Hot Spot | Non-Hot Spot | Non-Hot Spot | 38.9094 |
| **149** | Warren County, GA | 13301 | Hot Spot | Non-Hot Spot | Non-Hot Spot | 42.9611 |
| **150** | Washington County, GA | 13303 | Non-Hot Spot | Non-Hot Spot | Non-Hot Spot | 34.5260 |
| **151** | Wayne County, GA | 13305 | Non-Hot Spot | Non-Hot Spot | Non-Hot Spot | 32.8961 |
| **152** | Webster County, GA | 13307 | Non-Hot Spot | Non-Hot Spot | Non-Hot Spot | 32.1420 |
| **153** | Wheeler County, GA | 13309 | Non-Hot Spot | Non-Hot Spot | Non-Hot Spot | 36.5086 |
| **154** | White County, GA | 13311 | Non-Hot Spot | Non-Hot Spot | Non-Hot Spot | 32.8000 |
| **155** | Whitfield County, GA | 13313 | Non-Hot Spot | Non-Hot Spot | Non-Hot Spot | 31.2604 |
| **156** | Wilcox County, GA | 13315 | Non-Hot Spot | Non-Hot Spot | Non-Hot Spot | 38.3487 |
| **157** | Wilkes County, GA | 13317 | Hot Spot | Non-Hot Spot | Non-Hot Spot | 51.8729 |
| **158** | Wilkinson County, GA | 13319 | Non-Hot Spot | Non-Hot Spot | Non-Hot Spot | 32.3703 |
| **159** | Worth County, GA | 13321 | Non-Hot Spot | Non-Hot Spot | Non-Hot Spot | 32.8047 |
