# Supplementary material for: Understanding geographic and racial/ethnic disparities in mortality from four major cancers in the state of Georgia: a spatial epidemiologic analysis, 1999–2019
Source: Sci Rep. 2022 Aug 19;12:14143. doi: 10.1038/s41598-022-18374-7 (PMC9391349; doi:10.1038/s41598-022-18374-7)
Supplement: Supplementary file 17 — Supplementary Information 17. [file 41598_2022_18374_MOESM17_ESM.docx]

| **Obs** | **County** | **GEOID** | **Colorectal Cancer Mortality Hot Spots for All Adults** | **Colorectal Cancer Mortality Hot Spots for African American Adults** | **Colorectal Cancer Mortality Hot Spots for NH-White Adults** | **Empirical Bayes Smoothed Mortality Rate, All Adults per 100,000** |
| --- | --- | --- | --- | --- | --- | --- |
| **1** | Appling County, GA | 13001 | Non-Hot Spot | Non-Hot Spot | Hot Spot | 27.3454 |
| **2** | Atkinson County, GA | 13003 | Non-Hot Spot | Non-Hot Spot | Non-Hot Spot | 22.5640 |
| **3** | Bacon County, GA | 13005 | Non-Hot Spot | Non-Hot Spot | Non-Hot Spot | 26.4489 |
| **4** | Baker County, GA | 13007 | Non-Hot Spot | Non-Hot Spot | Non-Hot Spot | 26.3131 |
| **5** | Baldwin County, GA | 13009 | Non-Hot Spot | Non-Hot Spot | Non-Hot Spot | 19.9557 |
| **6** | Banks County, GA | 13011 | Non-Hot Spot | Non-Hot Spot | Non-Hot Spot | 23.6286 |
| **7** | Barrow County, GA | 13013 | Non-Hot Spot | Non-Hot Spot | Non-Hot Spot | 18.8791 |
| **8** | Bartow County, GA | 13015 | Non-Hot Spot | Non-Hot Spot | Non-Hot Spot | 19.7764 |
| **9** | Ben Hill County, GA | 13017 | Non-Hot Spot | Non-Hot Spot | Non-Hot Spot | 30.8901 |
| **10** | Berrien County, GA | 13019 | Non-Hot Spot | Non-Hot Spot | Non-Hot Spot | 20.6407 |
| **11** | Bibb County, GA | 13021 | Non-Hot Spot | Non-Hot Spot | Non-Hot Spot | 27.1769 |
| **12** | Bleckley County, GA | 13023 | Non-Hot Spot | Non-Hot Spot | Non-Hot Spot | 22.2401 |
| **13** | Brantley County, GA | 13025 | Non-Hot Spot | Non-Hot Spot | Non-Hot Spot | 19.6770 |
| **14** | Brooks County, GA | 13027 | Non-Hot Spot | Non-Hot Spot | Non-Hot Spot | 26.2407 |
| **15** | Bryan County, GA | 13029 | Non-Hot Spot | Non-Hot Spot | Non-Hot Spot | 22.2975 |
| **16** | Bulloch County, GA | 13031 | Non-Hot Spot | Non-Hot Spot | Non-Hot Spot | 16.2792 |
| **17** | Burke County, GA | 13033 | Hot Spot | Hot Spot | Non-Hot Spot | 30.6574 |
| **18** | Butts County, GA | 13035 | Non-Hot Spot | Non-Hot Spot | Non-Hot Spot | 24.5771 |
| **19** | Calhoun County, GA | 13037 | Non-Hot Spot | Non-Hot Spot | Non-Hot Spot | 20.0008 |
| **20** | Camden County, GA | 13039 | Non-Hot Spot | Non-Hot Spot | Non-Hot Spot | 17.4498 |
| **21** | Candler County, GA | 13043 | Non-Hot Spot | Non-Hot Spot | Hot Spot | 27.4558 |
| **22** | Carroll County, GA | 13045 | Non-Hot Spot | Non-Hot Spot | Non-Hot Spot | 22.0511 |
| **23** | Catoosa County, GA | 13047 | Non-Hot Spot | Non-Hot Spot | Non-Hot Spot | 19.2738 |
| **24** | Charlton County, GA | 13049 | Non-Hot Spot | Non-Hot Spot | Non-Hot Spot | 19.4124 |
| **25** | Chatham County, GA | 13051 | Non-Hot Spot | Non-Hot Spot | Non-Hot Spot | 23.5386 |
| **26** | Chattahoochee County, | 13053 | Non-Hot Spot | Non-Hot Spot | Non-Hot Spot | 15.9240 |
| **27** | Chattooga County, GA | 13055 | Non-Hot Spot | Non-Hot Spot | Non-Hot Spot | 25.8983 |
| **28** | Cherokee County, GA | 13057 | Non-Hot Spot | Non-Hot Spot | Non-Hot Spot | 15.3278 |
| **29** | Clarke County, GA | 13059 | Non-Hot Spot | Non-Hot Spot | Non-Hot Spot | 12.8122 |
| **30** | Clay County, GA | 13061 | Non-Hot Spot | Non-Hot Spot | Non-Hot Spot | 34.0096 |
| **31** | Clayton County, GA | 13063 | Non-Hot Spot | Non-Hot Spot | Non-Hot Spot | 16.2602 |
| **32** | Clinch County, GA | 13065 | Non-Hot Spot | Non-Hot Spot | Non-Hot Spot | 22.5065 |
| **33** | Cobb County, GA | 13067 | Non-Hot Spot | Non-Hot Spot | Non-Hot Spot | 15.3613 |
| **34** | Coffee County, GA | 13069 | Non-Hot Spot | Non-Hot Spot | Non-Hot Spot | 21.3573 |
| **35** | Colquitt County, GA | 13071 | Non-Hot Spot | Non-Hot Spot | Non-Hot Spot | 22.9700 |
| **36** | Columbia County, GA | 13073 | Non-Hot Spot | Non-Hot Spot | Non-Hot Spot | 14.7915 |
| **37** | Cook County, GA | 13075 | Non-Hot Spot | Non-Hot Spot | Non-Hot Spot | 24.5630 |
| **38** | Coweta County, GA | 13077 | Non-Hot Spot | Non-Hot Spot | Non-Hot Spot | 18.0029 |
| **39** | Crawford County, GA | 13079 | Non-Hot Spot | Non-Hot Spot | Non-Hot Spot | 23.1913 |
| **40** | Crisp County, GA | 13081 | Non-Hot Spot | Non-Hot Spot | Non-Hot Spot | 25.8381 |
| **41** | Dade County, GA | 13083 | Non-Hot Spot | Non-Hot Spot | Non-Hot Spot | 26.3781 |
| **42** | Dawson County, GA | 13085 | Non-Hot Spot | Non-Hot Spot | Non-Hot Spot | 22.0999 |
| **43** | Decatur County, GA | 13087 | Non-Hot Spot | Non-Hot Spot | Non-Hot Spot | 29.0524 |
| **44** | DeKalb County, GA | 13089 | Non-Hot Spot | Non-Hot Spot | Non-Hot Spot | 16.7141 |
| **45** | Dodge County, GA | 13091 | Non-Hot Spot | Non-Hot Spot | Non-Hot Spot | 25.9402 |
| **46** | Dooly County, GA | 13093 | Non-Hot Spot | Non-Hot Spot | Non-Hot Spot | 20.8440 |
| **47** | Dougherty County, GA | 13095 | Non-Hot Spot | Non-Hot Spot | Non-Hot Spot | 21.8174 |
| **48** | Douglas County, GA | 13097 | Non-Hot Spot | Non-Hot Spot | Non-Hot Spot | 17.2259 |
| **49** | Early County, GA | 13099 | Non-Hot Spot | Non-Hot Spot | Non-Hot Spot | 30.1612 |
| **50** | Echols County, GA | 13101 | Non-Hot Spot | Non-Hot Spot | Non-Hot Spot | 10.9464 |
| **51** | Effingham County, GA | 13103 | Non-Hot Spot | Non-Hot Spot | Non-Hot Spot | 22.3445 |
| **52** | Elbert County, GA | 13105 | Hot Spot | Hot Spot | Non-Hot Spot | 29.0369 |
| **53** | Emanuel County, GA | 13107 | Non-Hot Spot | Non-Hot Spot | Non-Hot Spot | 34.7108 |
| **54** | Evans County, GA | 13109 | Non-Hot Spot | Non-Hot Spot | Non-Hot Spot | 25.1833 |
| **55** | Fannin County, GA | 13111 | Non-Hot Spot | Non-Hot Spot | Non-Hot Spot | 35.1386 |
| **56** | Fayette County, GA | 13113 | Non-Hot Spot | Non-Hot Spot | Non-Hot Spot | 19.8596 |
| **57** | Floyd County, GA | 13115 | Non-Hot Spot | Non-Hot Spot | Non-Hot Spot | 24.0201 |
| **58** | Forsyth County, GA | 13117 | Non-Hot Spot | Non-Hot Spot | Non-Hot Spot | 15.3527 |
| **59** | Franklin County, GA | 13119 | Hot Spot | Non-Hot Spot | Hot Spot | 34.8791 |
| **60** | Fulton County, GA | 13121 | Non-Hot Spot | Non-Hot Spot | Non-Hot Spot | 17.8876 |
| **61** | Gilmer County, GA | 13123 | Non-Hot Spot | Non-Hot Spot | Non-Hot Spot | 22.6099 |
| **62** | Glascock County, GA | 13125 | Non-Hot Spot | Hot Spot | Non-Hot Spot | 29.6009 |
| **63** | Glynn County, GA | 13127 | Non-Hot Spot | Non-Hot Spot | Non-Hot Spot | 23.2272 |
| **64** | Gordon County, GA | 13129 | Non-Hot Spot | Non-Hot Spot | Non-Hot Spot | 20.9803 |
| **65** | Grady County, GA | 13131 | Non-Hot Spot | Non-Hot Spot | Non-Hot Spot | 28.3570 |
| **66** | Greene County, GA | 13133 | Non-Hot Spot | Non-Hot Spot | Non-Hot Spot | 34.2910 |
| **67** | Gwinnett County, GA | 13135 | Non-Hot Spot | Non-Hot Spot | Non-Hot Spot | 12.8641 |
| **68** | Habersham County, GA | 13137 | Non-Hot Spot | Non-Hot Spot | Non-Hot Spot | 23.5025 |
| **69** | Hall County, GA | 13139 | Non-Hot Spot | Non-Hot Spot | Non-Hot Spot | 18.2225 |
| **70** | Hancock County, GA | 13141 | Non-Hot Spot | Non-Hot Spot | Non-Hot Spot | 26.9133 |
| **71** | Haralson County, GA | 13143 | Non-Hot Spot | Non-Hot Spot | Non-Hot Spot | 27.5314 |
| **72** | Harris County, GA | 13145 | Non-Hot Spot | Non-Hot Spot | Non-Hot Spot | 25.3902 |
| **73** | Hart County, GA | 13147 | Non-Hot Spot | Non-Hot Spot | Hot Spot | 32.4158 |
| **74** | Heard County, GA | 13149 | Non-Hot Spot | Non-Hot Spot | Non-Hot Spot | 21.9931 |
| **75** | Henry County, GA | 13151 | Non-Hot Spot | Non-Hot Spot | Non-Hot Spot | 18.2804 |
| **76** | Houston County, GA | 13153 | Non-Hot Spot | Non-Hot Spot | Non-Hot Spot | 18.8217 |
| **77** | Irwin County, GA | 13155 | Non-Hot Spot | Non-Hot Spot | Non-Hot Spot | 26.8961 |
| **78** | Jackson County, GA | 13157 | Non-Hot Spot | Non-Hot Spot | Non-Hot Spot | 22.2223 |
| **79** | Jasper County, GA | 13159 | Non-Hot Spot | Non-Hot Spot | Non-Hot Spot | 24.0486 |
| **80** | Jeff Davis County, GA | 13161 | Non-Hot Spot | Non-Hot Spot | Non-Hot Spot | 25.8029 |
| **81** | Jefferson County, GA | 13163 | Hot Spot | Non-Hot Spot | Hot Spot | 36.9501 |
| **82** | Jenkins County, GA | 13165 | Hot Spot | Non-Hot Spot | Hot Spot | 32.2692 |
| **83** | Johnson County, GA | 13167 | Non-Hot Spot | Non-Hot Spot | Non-Hot Spot | 24.2172 |
| **84** | Jones County, GA | 13169 | Non-Hot Spot | Non-Hot Spot | Non-Hot Spot | 22.4847 |
| **85** | Lamar County, GA | 13171 | Non-Hot Spot | Non-Hot Spot | Non-Hot Spot | 25.9793 |
| **86** | Lanier County, GA | 13173 | Non-Hot Spot | Non-Hot Spot | Non-Hot Spot | 20.1613 |
| **87** | Laurens County, GA | 13175 | Non-Hot Spot | Non-Hot Spot | Non-Hot Spot | 27.8084 |
| **88** | Lee County, GA | 13177 | Non-Hot Spot | Non-Hot Spot | Non-Hot Spot | 18.3184 |
| **89** | Liberty County, GA | 13179 | Non-Hot Spot | Non-Hot Spot | Non-Hot Spot | 15.3916 |
| **90** | Lincoln County, GA | 13181 | Non-Hot Spot | Non-Hot Spot | Non-Hot Spot | 25.2721 |
| **91** | Long County, GA | 13183 | Non-Hot Spot | Non-Hot Spot | Non-Hot Spot | 14.5877 |
| **92** | Lowndes County, GA | 13185 | Non-Hot Spot | Non-Hot Spot | Non-Hot Spot | 15.0903 |
| **93** | Lumpkin County, GA | 13187 | Non-Hot Spot | Non-Hot Spot | Non-Hot Spot | 18.5868 |
| **94** | McDuffie County, GA | 13189 | Hot Spot | Non-Hot Spot | Non-Hot Spot | 30.8644 |
| **95** | McIntosh County, GA | 13191 | Non-Hot Spot | Non-Hot Spot | Non-Hot Spot | 22.7412 |
| **96** | Macon County, GA | 13193 | Non-Hot Spot | Non-Hot Spot | Non-Hot Spot | 25.2921 |
| **97** | Madison County, GA | 13195 | Non-Hot Spot | Non-Hot Spot | Non-Hot Spot | 26.2836 |
| **98** | Marion County, GA | 13197 | Non-Hot Spot | Non-Hot Spot | Non-Hot Spot | 27.7997 |
| **99** | Meriwether County, GA | 13199 | Non-Hot Spot | Non-Hot Spot | Non-Hot Spot | 26.6685 |
| **100** | Miller County, GA | 13201 | Non-Hot Spot | Non-Hot Spot | Non-Hot Spot | 27.9633 |
| **101** | Mitchell County, GA | 13205 | Non-Hot Spot | Non-Hot Spot | Non-Hot Spot | 26.3747 |
| **102** | Monroe County, GA | 13207 | Non-Hot Spot | Non-Hot Spot | Non-Hot Spot | 25.3459 |
| **103** | Montgomery County, GA | 13209 | Non-Hot Spot | Non-Hot Spot | Non-Hot Spot | 25.3639 |
| **104** | Morgan County, GA | 13211 | Non-Hot Spot | Non-Hot Spot | Non-Hot Spot | 25.4306 |
| **105** | Murray County, GA | 13213 | Non-Hot Spot | Non-Hot Spot | Non-Hot Spot | 18.9960 |
| **106** | Muscogee County, GA | 13215 | Non-Hot Spot | Non-Hot Spot | Non-Hot Spot | 25.3357 |
| **107** | Newton County, GA | 13217 | Non-Hot Spot | Non-Hot Spot | Non-Hot Spot | 20.3138 |
| **108** | Oconee County, GA | 13219 | Non-Hot Spot | Non-Hot Spot | Non-Hot Spot | 16.4908 |
| **109** | Oglethorpe County, GA | 13221 | Non-Hot Spot | Non-Hot Spot | Non-Hot Spot | 25.2899 |
| **110** | Paulding County, GA | 13223 | Non-Hot Spot | Non-Hot Spot | Non-Hot Spot | 15.5706 |
| **111** | Peach County, GA | 13225 | Non-Hot Spot | Non-Hot Spot | Non-Hot Spot | 28.7966 |
| **112** | Pickens County, GA | 13227 | Non-Hot Spot | Non-Hot Spot | Non-Hot Spot | 19.2796 |
| **113** | Pierce County, GA | 13229 | Non-Hot Spot | Non-Hot Spot | Non-Hot Spot | 26.9827 |
| **114** | Pike County, GA | 13231 | Non-Hot Spot | Non-Hot Spot | Non-Hot Spot | 28.1960 |
| **115** | Polk County, GA | 13233 | Non-Hot Spot | Non-Hot Spot | Non-Hot Spot | 23.0191 |
| **116** | Pulaski County, GA | 13235 | Non-Hot Spot | Non-Hot Spot | Non-Hot Spot | 20.9543 |
| **117** | Putnam County, GA | 13237 | Non-Hot Spot | Hot Spot | Non-Hot Spot | 27.1571 |
| **118** | Quitman County, GA | 13239 | Hot Spot | Non-Hot Spot | Non-Hot Spot | 37.4045 |
| **119** | Rabun County, GA | 13241 | Non-Hot Spot | Non-Hot Spot | Non-Hot Spot | 28.2828 |
| **120** | Randolph County, GA | 13243 | Hot Spot | Hot Spot | Non-Hot Spot | 37.2535 |
| **121** | Richmond County, GA | 13245 | Non-Hot Spot | Non-Hot Spot | Non-Hot Spot | 22.4708 |
| **122** | Rockdale County, GA | 13247 | Non-Hot Spot | Non-Hot Spot | Non-Hot Spot | 19.8650 |
| **123** | Schley County, GA | 13249 | Non-Hot Spot | Non-Hot Spot | Non-Hot Spot | 24.4525 |
| **124** | Screven County, GA | 13251 | Hot Spot | Non-Hot Spot | Hot Spot | 34.1427 |
| **125** | Seminole County, GA | 13253 | Non-Hot Spot | Non-Hot Spot | Non-Hot Spot | 30.9796 |
| **126** | Spalding County, GA | 13255 | Non-Hot Spot | Non-Hot Spot | Non-Hot Spot | 27.4932 |
| **127** | Stephens County, GA | 13257 | Non-Hot Spot | Non-Hot Spot | Non-Hot Spot | 38.1003 |
| **128** | Stewart County, GA | 13259 | Non-Hot Spot | Non-Hot Spot | Non-Hot Spot | 33.5975 |
| **129** | Sumter County, GA | 13261 | Non-Hot Spot | Non-Hot Spot | Non-Hot Spot | 21.1043 |
| **130** | Talbot County, GA | 13263 | Non-Hot Spot | Non-Hot Spot | Non-Hot Spot | 27.7853 |
| **131** | Taliaferro County, GA | 13265 | Hot Spot | Non-Hot Spot | Non-Hot Spot | 36.0961 |
| **132** | Tattnall County, GA | 13267 | Non-Hot Spot | Non-Hot Spot | Non-Hot Spot | 24.3274 |
| **133** | Taylor County, GA | 13269 | Non-Hot Spot | Non-Hot Spot | Non-Hot Spot | 31.9526 |
| **134** | Telfair County, GA | 13271 | Non-Hot Spot | Non-Hot Spot | Non-Hot Spot | 22.8767 |
| **135** | Terrell County, GA | 13273 | Non-Hot Spot | Non-Hot Spot | Non-Hot Spot | 44.3242 |
| **136** | Thomas County, GA | 13275 | Non-Hot Spot | Non-Hot Spot | Non-Hot Spot | 26.8511 |
| **137** | Tift County, GA | 13277 | Non-Hot Spot | Non-Hot Spot | Non-Hot Spot | 21.3935 |
| **138** | Toombs County, GA | 13279 | Non-Hot Spot | Non-Hot Spot | Non-Hot Spot | 26.8555 |
| **139** | Towns County, GA | 13281 | Non-Hot Spot | Non-Hot Spot | Non-Hot Spot | 27.5284 |
| **140** | Treutlen County, GA | 13283 | Non-Hot Spot | Non-Hot Spot | Non-Hot Spot | 25.2688 |
| **141** | Troup County, GA | 13285 | Non-Hot Spot | Non-Hot Spot | Non-Hot Spot | 29.2760 |
| **142** | Turner County, GA | 13287 | Non-Hot Spot | Non-Hot Spot | Non-Hot Spot | 29.3848 |
| **143** | Twiggs County, GA | 13289 | Non-Hot Spot | Non-Hot Spot | Non-Hot Spot | 27.8905 |
| **144** | Union County, GA | 13291 | Non-Hot Spot | Non-Hot Spot | Non-Hot Spot | 29.0941 |
| **145** | Upson County, GA | 13293 | Non-Hot Spot | Non-Hot Spot | Non-Hot Spot | 30.8535 |
| **146** | Walker County, GA | 13295 | Non-Hot Spot | Non-Hot Spot | Non-Hot Spot | 25.3436 |
| **147** | Walton County, GA | 13297 | Non-Hot Spot | Non-Hot Spot | Non-Hot Spot | 23.1116 |
| **148** | Ware County, GA | 13299 | Non-Hot Spot | Non-Hot Spot | Non-Hot Spot | 24.7378 |
| **149** | Warren County, GA | 13301 | Hot Spot | Non-Hot Spot | Non-Hot Spot | 36.8475 |
| **150** | Washington County, GA | 13303 | Non-Hot Spot | Non-Hot Spot | Non-Hot Spot | 24.6858 |
| **151** | Wayne County, GA | 13305 | Non-Hot Spot | Non-Hot Spot | Non-Hot Spot | 26.6656 |
| **152** | Webster County, GA | 13307 | Hot Spot | Hot Spot | Non-Hot Spot | 36.1058 |
| **153** | Wheeler County, GA | 13309 | Non-Hot Spot | Non-Hot Spot | Non-Hot Spot | 25.7739 |
| **154** | White County, GA | 13311 | Non-Hot Spot | Non-Hot Spot | Non-Hot Spot | 21.7556 |
| **155** | Whitfield County, GA | 13313 | Non-Hot Spot | Non-Hot Spot | Non-Hot Spot | 17.7618 |
| **156** | Wilcox County, GA | 13315 | Non-Hot Spot | Non-Hot Spot | Non-Hot Spot | 29.2612 |
| **157** | Wilkes County, GA | 13317 | Hot Spot | Non-Hot Spot | Non-Hot Spot | 36.4998 |
| **158** | Wilkinson County, GA | 13319 | Non-Hot Spot | Non-Hot Spot | Non-Hot Spot | 25.1476 |
| **159** | Worth County, GA | 13321 | Non-Hot Spot | Non-Hot Spot | Non-Hot Spot | 20.3091 |
