# Supplementary material for: Understanding geographic and racial/ethnic disparities in mortality from four major cancers in the state of Georgia: a spatial epidemiologic analysis, 1999–2019
Source: Sci Rep. 2022 Aug 19;12:14143. doi: 10.1038/s41598-022-18374-7 (PMC9391349; doi:10.1038/s41598-022-18374-7)
Supplement: Supplementary file 18 — Supplementary Information 18. [file 41598_2022_18374_MOESM18_ESM.docx]

| **Obs** | **County** | **GEOID** | **Lung Cancer Mortality Hot Spots for All Adults** | **Lung Cancer Mortality Hot Spots for African American Adults** | **Lung Cancer Mortality Hot Spots for NH-White Adults** | **Empirical Bayes Smoothed Mortality Rate, All Adults per 100,000** |
| --- | --- | --- | --- | --- | --- | --- |
| **1** | Appling County, GA | 13001 | Non-Hot Spot | Non-Hot Spot | Non-Hot Spot | 86.181 |
| **2** | Atkinson County, GA | 13003 | Non-Hot Spot | Non-Hot Spot | Non-Hot Spot | 88.537 |
| **3** | Bacon County, GA | 13005 | Non-Hot Spot | Non-Hot Spot | Non-Hot Spot | 97.281 |
| **4** | Baker County, GA | 13007 | Non-Hot Spot | Non-Hot Spot | Non-Hot Spot | 82.287 |
| **5** | Baldwin County, GA | 13009 | Non-Hot Spot | Non-Hot Spot | Non-Hot Spot | 70.542 |
| **6** | Banks County, GA | 13011 | Non-Hot Spot | Non-Hot Spot | Non-Hot Spot | 78.714 |
| **7** | Barrow County, GA | 13013 | Non-Hot Spot | Non-Hot Spot | Non-Hot Spot | 71.851 |
| **8** | Bartow County, GA | 13015 | Non-Hot Spot | Non-Hot Spot | Non-Hot Spot | 78.056 |
| **9** | Ben Hill County, GA | 13017 | Non-Hot Spot | Non-Hot Spot | Non-Hot Spot | 107.440 |
| **10** | Berrien County, GA | 13019 | Non-Hot Spot | Non-Hot Spot | Non-Hot Spot | 91.996 |
| **11** | Bibb County, GA | 13021 | Non-Hot Spot | Non-Hot Spot | Non-Hot Spot | 79.828 |
| **12** | Bleckley County, GA | 13023 | Non-Hot Spot | Non-Hot Spot | Non-Hot Spot | 92.246 |
| **13** | Brantley County, GA | 13025 | Non-Hot Spot | Non-Hot Spot | Non-Hot Spot | 112.308 |
| **14** | Brooks County, GA | 13027 | Non-Hot Spot | Non-Hot Spot | Non-Hot Spot | 94.070 |
| **15** | Bryan County, GA | 13029 | Non-Hot Spot | Non-Hot Spot | Non-Hot Spot | 66.720 |
| **16** | Bulloch County, GA | 13031 | Non-Hot Spot | Non-Hot Spot | Non-Hot Spot | 53.168 |
| **17** | Burke County, GA | 13033 | Non-Hot Spot | Non-Hot Spot | Non-Hot Spot | 81.602 |
| **18** | Butts County, GA | 13035 | Non-Hot Spot | Non-Hot Spot | Non-Hot Spot | 96.523 |
| **19** | Calhoun County, GA | 13037 | Non-Hot Spot | Non-Hot Spot | Hot Spot | 75.153 |
| **20** | Camden County, GA | 13039 | Non-Hot Spot | Non-Hot Spot | Non-Hot Spot | 53.405 |
| **21** | Candler County, GA | 13043 | Non-Hot Spot | Non-Hot Spot | Non-Hot Spot | 82.640 |
| **22** | Carroll County, GA | 13045 | Non-Hot Spot | Non-Hot Spot | Non-Hot Spot | 72.610 |
| **23** | Catoosa County, GA | 13047 | Non-Hot Spot | Non-Hot Spot | Non-Hot Spot | 85.675 |
| **24** | Charlton County, GA | 13049 | Non-Hot Spot | Non-Hot Spot | Non-Hot Spot | 78.196 |
| **25** | Chatham County, GA | 13051 | Non-Hot Spot | Non-Hot Spot | Non-Hot Spot | 64.419 |
| **26** | Chattahoochee County, | 13053 | Non-Hot Spot | Non-Hot Spot | Non-Hot Spot | 29.314 |
| **27** | Chattooga County, GA | 13055 | Hot Spot | Hot Spot | Non-Hot Spot | 104.743 |
| **28** | Cherokee County, GA | 13057 | Non-Hot Spot | Non-Hot Spot | Non-Hot Spot | 50.054 |
| **29** | Clarke County, GA | 13059 | Non-Hot Spot | Non-Hot Spot | Non-Hot Spot | 36.857 |
| **30** | Clay County, GA | 13061 | Hot Spot | Non-Hot Spot | Hot Spot | 104.762 |
| **31** | Clayton County, GA | 13063 | Non-Hot Spot | Non-Hot Spot | Non-Hot Spot | 44.364 |
| **32** | Clinch County, GA | 13065 | Non-Hot Spot | Non-Hot Spot | Non-Hot Spot | 95.428 |
| **33** | Cobb County, GA | 13067 | Non-Hot Spot | Non-Hot Spot | Non-Hot Spot | 41.703 |
| **34** | Coffee County, GA | 13069 | Non-Hot Spot | Non-Hot Spot | Non-Hot Spot | 77.556 |
| **35** | Colquitt County, GA | 13071 | Non-Hot Spot | Non-Hot Spot | Non-Hot Spot | 91.628 |
| **36** | Columbia County, GA | 13073 | Non-Hot Spot | Non-Hot Spot | Non-Hot Spot | 56.913 |
| **37** | Cook County, GA | 13075 | Non-Hot Spot | Non-Hot Spot | Non-Hot Spot | 83.143 |
| **38** | Coweta County, GA | 13077 | Non-Hot Spot | Non-Hot Spot | Non-Hot Spot | 59.960 |
| **39** | Crawford County, GA | 13079 | Non-Hot Spot | Non-Hot Spot | Non-Hot Spot | 90.407 |
| **40** | Crisp County, GA | 13081 | Non-Hot Spot | Non-Hot Spot | Non-Hot Spot | 91.170 |
| **41** | Dade County, GA | 13083 | Hot Spot | Non-Hot Spot | Non-Hot Spot | 115.933 |
| **42** | Dawson County, GA | 13085 | Non-Hot Spot | Non-Hot Spot | Non-Hot Spot | 82.545 |
| **43** | Decatur County, GA | 13087 | Non-Hot Spot | Non-Hot Spot | Non-Hot Spot | 90.200 |
| **44** | DeKalb County, GA | 13089 | Non-Hot Spot | Non-Hot Spot | Non-Hot Spot | 39.528 |
| **45** | Dodge County, GA | 13091 | Non-Hot Spot | Non-Hot Spot | Non-Hot Spot | 86.235 |
| **46** | Dooly County, GA | 13093 | Non-Hot Spot | Non-Hot Spot | Non-Hot Spot | 66.030 |
| **47** | Dougherty County, GA | 13095 | Non-Hot Spot | Non-Hot Spot | Non-Hot Spot | 82.785 |
| **48** | Douglas County, GA | 13097 | Non-Hot Spot | Non-Hot Spot | Non-Hot Spot | 56.497 |
| **49** | Early County, GA | 13099 | Non-Hot Spot | Non-Hot Spot | Hot Spot | 101.529 |
| **50** | Echols County, GA | 13101 | Non-Hot Spot | Non-Hot Spot | Non-Hot Spot | 63.915 |
| **51** | Effingham County, GA | 13103 | Non-Hot Spot | Non-Hot Spot | Non-Hot Spot | 65.851 |
| **52** | Elbert County, GA | 13105 | Non-Hot Spot | Hot Spot | Non-Hot Spot | 100.036 |
| **53** | Emanuel County, GA | 13107 | Non-Hot Spot | Non-Hot Spot | Non-Hot Spot | 90.124 |
| **54** | Evans County, GA | 13109 | Non-Hot Spot | Non-Hot Spot | Non-Hot Spot | 79.803 |
| **55** | Fannin County, GA | 13111 | Non-Hot Spot | Non-Hot Spot | Non-Hot Spot | 108.105 |
| **56** | Fayette County, GA | 13113 | Non-Hot Spot | Non-Hot Spot | Non-Hot Spot | 49.677 |
| **57** | Floyd County, GA | 13115 | Non-Hot Spot | Hot Spot | Non-Hot Spot | 93.439 |
| **58** | Forsyth County, GA | 13117 | Non-Hot Spot | Non-Hot Spot | Non-Hot Spot | 41.885 |
| **59** | Franklin County, GA | 13119 | Non-Hot Spot | Non-Hot Spot | Non-Hot Spot | 97.340 |
| **60** | Fulton County, GA | 13121 | Non-Hot Spot | Non-Hot Spot | Non-Hot Spot | 43.328 |
| **61** | Gilmer County, GA | 13123 | Non-Hot Spot | Non-Hot Spot | Non-Hot Spot | 83.150 |
| **62** | Glascock County, GA | 13125 | Non-Hot Spot | Hot Spot | Hot Spot | 95.696 |
| **63** | Glynn County, GA | 13127 | Non-Hot Spot | Non-Hot Spot | Non-Hot Spot | 85.503 |
| **64** | Gordon County, GA | 13129 | Non-Hot Spot | Non-Hot Spot | Non-Hot Spot | 91.404 |
| **65** | Grady County, GA | 13131 | Non-Hot Spot | Non-Hot Spot | Non-Hot Spot | 91.446 |
| **66** | Greene County, GA | 13133 | Non-Hot Spot | Non-Hot Spot | Non-Hot Spot | 79.697 |
| **67** | Gwinnett County, GA | 13135 | Non-Hot Spot | Non-Hot Spot | Non-Hot Spot | 34.072 |
| **68** | Habersham County, GA | 13137 | Non-Hot Spot | Non-Hot Spot | Non-Hot Spot | 79.599 |
| **69** | Hall County, GA | 13139 | Non-Hot Spot | Non-Hot Spot | Non-Hot Spot | 60.475 |
| **70** | Hancock County, GA | 13141 | Non-Hot Spot | Non-Hot Spot | Non-Hot Spot | 84.335 |
| **71** | Haralson County, GA | 13143 | Non-Hot Spot | Non-Hot Spot | Non-Hot Spot | 102.686 |
| **72** | Harris County, GA | 13145 | Non-Hot Spot | Non-Hot Spot | Non-Hot Spot | 67.148 |
| **73** | Hart County, GA | 13147 | Non-Hot Spot | Non-Hot Spot | Non-Hot Spot | 94.418 |
| **74** | Heard County, GA | 13149 | Non-Hot Spot | Non-Hot Spot | Non-Hot Spot | 88.429 |
| **75** | Henry County, GA | 13151 | Non-Hot Spot | Non-Hot Spot | Non-Hot Spot | 52.476 |
| **76** | Houston County, GA | 13153 | Non-Hot Spot | Non-Hot Spot | Non-Hot Spot | 66.054 |
| **77** | Irwin County, GA | 13155 | Non-Hot Spot | Non-Hot Spot | Non-Hot Spot | 90.835 |
| **78** | Jackson County, GA | 13157 | Non-Hot Spot | Non-Hot Spot | Non-Hot Spot | 79.790 |
| **79** | Jasper County, GA | 13159 | Non-Hot Spot | Non-Hot Spot | Non-Hot Spot | 77.794 |
| **80** | Jeff Davis County, GA | 13161 | Non-Hot Spot | Non-Hot Spot | Non-Hot Spot | 95.217 |
| **81** | Jefferson County, GA | 13163 | Non-Hot Spot | Non-Hot Spot | Non-Hot Spot | 94.241 |
| **82** | Jenkins County, GA | 13165 | Non-Hot Spot | Non-Hot Spot | Non-Hot Spot | 80.728 |
| **83** | Johnson County, GA | 13167 | Non-Hot Spot | Non-Hot Spot | Non-Hot Spot | 89.113 |
| **84** | Jones County, GA | 13169 | Non-Hot Spot | Non-Hot Spot | Non-Hot Spot | 78.142 |
| **85** | Lamar County, GA | 13171 | Non-Hot Spot | Non-Hot Spot | Non-Hot Spot | 84.784 |
| **86** | Lanier County, GA | 13173 | Non-Hot Spot | Non-Hot Spot | Non-Hot Spot | 76.193 |
| **87** | Laurens County, GA | 13175 | Non-Hot Spot | Non-Hot Spot | Non-Hot Spot | 87.287 |
| **88** | Lee County, GA | 13177 | Non-Hot Spot | Non-Hot Spot | Non-Hot Spot | 73.221 |
| **89** | Liberty County, GA | 13179 | Non-Hot Spot | Non-Hot Spot | Non-Hot Spot | 43.651 |
| **90** | Lincoln County, GA | 13181 | Non-Hot Spot | Non-Hot Spot | Non-Hot Spot | 101.747 |
| **91** | Long County, GA | 13183 | Non-Hot Spot | Non-Hot Spot | Non-Hot Spot | 49.504 |
| **92** | Lowndes County, GA | 13185 | Non-Hot Spot | Non-Hot Spot | Non-Hot Spot | 60.532 |
| **93** | Lumpkin County, GA | 13187 | Non-Hot Spot | Non-Hot Spot | Non-Hot Spot | 78.152 |
| **94** | McDuffie County, GA | 13189 | Non-Hot Spot | Hot Spot | Non-Hot Spot | 92.795 |
| **95** | McIntosh County, GA | 13191 | Non-Hot Spot | Non-Hot Spot | Non-Hot Spot | 86.911 |
| **96** | Macon County, GA | 13193 | Non-Hot Spot | Non-Hot Spot | Non-Hot Spot | 79.399 |
| **97** | Madison County, GA | 13195 | Non-Hot Spot | Non-Hot Spot | Non-Hot Spot | 94.384 |
| **98** | Marion County, GA | 13197 | Non-Hot Spot | Non-Hot Spot | Non-Hot Spot | 75.506 |
| **99** | Meriwether County, GA | 13199 | Non-Hot Spot | Non-Hot Spot | Non-Hot Spot | 82.360 |
| **100** | Miller County, GA | 13201 | Non-Hot Spot | Non-Hot Spot | Non-Hot Spot | 104.194 |
| **101** | Mitchell County, GA | 13205 | Non-Hot Spot | Non-Hot Spot | Non-Hot Spot | 93.015 |
| **102** | Monroe County, GA | 13207 | Non-Hot Spot | Non-Hot Spot | Non-Hot Spot | 74.058 |
| **103** | Montgomery County, GA | 13209 | Non-Hot Spot | Non-Hot Spot | Non-Hot Spot | 86.775 |
| **104** | Morgan County, GA | 13211 | Non-Hot Spot | Non-Hot Spot | Non-Hot Spot | 76.095 |
| **105** | Murray County, GA | 13213 | Non-Hot Spot | Non-Hot Spot | Non-Hot Spot | 100.264 |
| **106** | Muscogee County, GA | 13215 | Non-Hot Spot | Non-Hot Spot | Non-Hot Spot | 71.849 |
| **107** | Newton County, GA | 13217 | Non-Hot Spot | Non-Hot Spot | Non-Hot Spot | 69.792 |
| **108** | Oconee County, GA | 13219 | Non-Hot Spot | Non-Hot Spot | Non-Hot Spot | 47.034 |
| **109** | Oglethorpe County, GA | 13221 | Non-Hot Spot | Non-Hot Spot | Non-Hot Spot | 78.264 |
| **110** | Paulding County, GA | 13223 | Non-Hot Spot | Non-Hot Spot | Non-Hot Spot | 52.887 |
| **111** | Peach County, GA | 13225 | Non-Hot Spot | Non-Hot Spot | Non-Hot Spot | 83.001 |
| **112** | Pickens County, GA | 13227 | Non-Hot Spot | Non-Hot Spot | Non-Hot Spot | 88.997 |
| **113** | Pierce County, GA | 13229 | Hot Spot | Non-Hot Spot | Non-Hot Spot | 103.246 |
| **114** | Pike County, GA | 13231 | Non-Hot Spot | Non-Hot Spot | Non-Hot Spot | 86.494 |
| **115** | Polk County, GA | 13233 | Non-Hot Spot | Non-Hot Spot | Non-Hot Spot | 113.581 |
| **116** | Pulaski County, GA | 13235 | Non-Hot Spot | Non-Hot Spot | Non-Hot Spot | 78.960 |
| **117** | Putnam County, GA | 13237 | Non-Hot Spot | Non-Hot Spot | Non-Hot Spot | 88.645 |
| **118** | Quitman County, GA | 13239 | Non-Hot Spot | Non-Hot Spot | Hot Spot | 145.337 |
| **119** | Rabun County, GA | 13241 | Non-Hot Spot | Non-Hot Spot | Non-Hot Spot | 97.946 |
| **120** | Randolph County, GA | 13243 | Non-Hot Spot | Hot Spot | Non-Hot Spot | 88.671 |
| **121** | Richmond County, GA | 13245 | Non-Hot Spot | Non-Hot Spot | Non-Hot Spot | 78.455 |
| **122** | Rockdale County, GA | 13247 | Non-Hot Spot | Non-Hot Spot | Non-Hot Spot | 58.750 |
| **123** | Schley County, GA | 13249 | Non-Hot Spot | Non-Hot Spot | Non-Hot Spot | 77.561 |
| **124** | Screven County, GA | 13251 | Non-Hot Spot | Non-Hot Spot | Non-Hot Spot | 90.663 |
| **125** | Seminole County, GA | 13253 | Hot Spot | Non-Hot Spot | Hot Spot | 100.478 |
| **126** | Spalding County, GA | 13255 | Non-Hot Spot | Non-Hot Spot | Non-Hot Spot | 83.554 |
| **127** | Stephens County, GA | 13257 | Non-Hot Spot | Non-Hot Spot | Non-Hot Spot | 100.709 |
| **128** | Stewart County, GA | 13259 | Non-Hot Spot | Non-Hot Spot | Non-Hot Spot | 83.173 |
| **129** | Sumter County, GA | 13261 | Non-Hot Spot | Non-Hot Spot | Non-Hot Spot | 74.354 |
| **130** | Talbot County, GA | 13263 | Non-Hot Spot | Non-Hot Spot | Non-Hot Spot | 102.377 |
| **131** | Taliaferro County, GA | 13265 | Non-Hot Spot | Hot Spot | Non-Hot Spot | 91.335 |
| **132** | Tattnall County, GA | 13267 | Non-Hot Spot | Non-Hot Spot | Non-Hot Spot | 86.442 |
| **133** | Taylor County, GA | 13269 | Non-Hot Spot | Non-Hot Spot | Non-Hot Spot | 90.620 |
| **134** | Telfair County, GA | 13271 | Non-Hot Spot | Non-Hot Spot | Non-Hot Spot | 76.615 |
| **135** | Terrell County, GA | 13273 | Non-Hot Spot | Non-Hot Spot | Non-Hot Spot | 95.877 |
| **136** | Thomas County, GA | 13275 | Non-Hot Spot | Hot Spot | Non-Hot Spot | 95.674 |
| **137** | Tift County, GA | 13277 | Non-Hot Spot | Non-Hot Spot | Non-Hot Spot | 77.053 |
| **138** | Toombs County, GA | 13279 | Non-Hot Spot | Non-Hot Spot | Non-Hot Spot | 89.427 |
| **139** | Towns County, GA | 13281 | Non-Hot Spot | Non-Hot Spot | Non-Hot Spot | 118.477 |
| **140** | Treutlen County, GA | 13283 | Non-Hot Spot | Non-Hot Spot | Non-Hot Spot | 88.118 |
| **141** | Troup County, GA | 13285 | Non-Hot Spot | Non-Hot Spot | Non-Hot Spot | 74.871 |
| **142** | Turner County, GA | 13287 | Non-Hot Spot | Non-Hot Spot | Non-Hot Spot | 92.959 |
| **143** | Twiggs County, GA | 13289 | Non-Hot Spot | Non-Hot Spot | Non-Hot Spot | 91.807 |
| **144** | Union County, GA | 13291 | Hot Spot | Non-Hot Spot | Non-Hot Spot | 106.527 |
| **145** | Upson County, GA | 13293 | Non-Hot Spot | Non-Hot Spot | Non-Hot Spot | 94.798 |
| **146** | Walker County, GA | 13295 | Hot Spot | Non-Hot Spot | Hot Spot | 119.390 |
| **147** | Walton County, GA | 13297 | Non-Hot Spot | Non-Hot Spot | Non-Hot Spot | 72.217 |
| **148** | Ware County, GA | 13299 | Non-Hot Spot | Non-Hot Spot | Non-Hot Spot | 93.513 |
| **149** | Warren County, GA | 13301 | Non-Hot Spot | Hot Spot | Non-Hot Spot | 104.177 |
| **150** | Washington County, GA | 13303 | Non-Hot Spot | Non-Hot Spot | Non-Hot Spot | 83.725 |
| **151** | Wayne County, GA | 13305 | Non-Hot Spot | Non-Hot Spot | Non-Hot Spot | 86.148 |
| **152** | Webster County, GA | 13307 | Non-Hot Spot | Hot Spot | Non-Hot Spot | 83.497 |
| **153** | Wheeler County, GA | 13309 | Non-Hot Spot | Non-Hot Spot | Non-Hot Spot | 78.243 |
| **154** | White County, GA | 13311 | Non-Hot Spot | Non-Hot Spot | Non-Hot Spot | 91.054 |
| **155** | Whitfield County, GA | 13313 | Non-Hot Spot | Non-Hot Spot | Non-Hot Spot | 82.002 |
| **156** | Wilcox County, GA | 13315 | Non-Hot Spot | Non-Hot Spot | Non-Hot Spot | 81.042 |
| **157** | Wilkes County, GA | 13317 | Hot Spot | Hot Spot | Non-Hot Spot | 104.491 |
| **158** | Wilkinson County, GA | 13319 | Non-Hot Spot | Non-Hot Spot | Non-Hot Spot | 87.985 |
| **159** | Worth County, GA | 13321 | Non-Hot Spot | Non-Hot Spot | Non-Hot Spot | 88.656 |
