# Supplementary material for: Understanding geographic and racial/ethnic disparities in mortality from four major cancers in the state of Georgia: a spatial epidemiologic analysis, 1999–2019
Source: Sci Rep. 2022 Aug 19;12:14143. doi: 10.1038/s41598-022-18374-7 (PMC9391349; doi:10.1038/s41598-022-18374-7)
Supplement: Supplementary file 19 — Supplementary Information 19. [file 41598_2022_18374_MOESM19_ESM.docx]

| **Obs** | **County** | **GEOID** | **Prostate Cancer Mortality Hot Spots for All Men** | **Prostate Cancer Mortality Hot Spots for African American Men** | **Prostate Cancer Mortality Hot Spots for NH-White Men** | **Empirical Bayes Smoothed Mortality Rate, All Men per 100,000** |
| --- | --- | --- | --- | --- | --- | --- |
| **1** | Appling County, GA | 13001 | Non-Hot Spot | Non-Hot Spot | Non-Hot Spot | 28.4830 |
| **2** | Atkinson County, GA | 13003 | Non-Hot Spot | Non-Hot Spot | Non-Hot Spot | 26.3088 |
| **3** | Bacon County, GA | 13005 | Non-Hot Spot | Non-Hot Spot | Non-Hot Spot | 27.1620 |
| **4** | Baker County, GA | 13007 | Non-Hot Spot | Non-Hot Spot | Non-Hot Spot | 24.7439 |
| **5** | Baldwin County, GA | 13009 | Non-Hot Spot | Non-Hot Spot | Non-Hot Spot | 21.6387 |
| **6** | Banks County, GA | 13011 | Non-Hot Spot | Non-Hot Spot | Non-Hot Spot | 16.6246 |
| **7** | Barrow County, GA | 13013 | Non-Hot Spot | Non-Hot Spot | Non-Hot Spot | 17.8081 |
| **8** | Bartow County, GA | 13015 | Non-Hot Spot | Non-Hot Spot | Non-Hot Spot | 19.3013 |
| **9** | Ben Hill County, GA | 13017 | Non-Hot Spot | Non-Hot Spot | Non-Hot Spot | 30.6830 |
| **10** | Berrien County, GA | 13019 | Non-Hot Spot | Non-Hot Spot | Non-Hot Spot | 24.4023 |
| **11** | Bibb County, GA | 13021 | Non-Hot Spot | Non-Hot Spot | Non-Hot Spot | 36.6449 |
| **12** | Bleckley County, GA | 13023 | Non-Hot Spot | Non-Hot Spot | Non-Hot Spot | 28.8518 |
| **13** | Brantley County, GA | 13025 | Non-Hot Spot | Non-Hot Spot | Non-Hot Spot | 25.0105 |
| **14** | Brooks County, GA | 13027 | Non-Hot Spot | Non-Hot Spot | Non-Hot Spot | 38.6931 |
| **15** | Bryan County, GA | 13029 | Non-Hot Spot | Non-Hot Spot | Non-Hot Spot | 20.5810 |
| **16** | Bulloch County, GA | 13031 | Non-Hot Spot | Non-Hot Spot | Non-Hot Spot | 19.9769 |
| **17** | Burke County, GA | 13033 | Hot Spot | Hot Spot | Hot Spot | 41.8894 |
| **18** | Butts County, GA | 13035 | Non-Hot Spot | Non-Hot Spot | Non-Hot Spot | 23.5191 |
| **19** | Calhoun County, GA | 13037 | Hot Spot | Non-Hot Spot | Non-Hot Spot | 37.1477 |
| **20** | Camden County, GA | 13039 | Non-Hot Spot | Non-Hot Spot | Non-Hot Spot | 13.8961 |
| **21** | Candler County, GA | 13043 | Non-Hot Spot | Non-Hot Spot | Non-Hot Spot | 26.5193 |
| **22** | Carroll County, GA | 13045 | Non-Hot Spot | Non-Hot Spot | Non-Hot Spot | 21.1684 |
| **23** | Catoosa County, GA | 13047 | Non-Hot Spot | Non-Hot Spot | Non-Hot Spot | 24.3451 |
| **24** | Charlton County, GA | 13049 | Non-Hot Spot | Non-Hot Spot | Non-Hot Spot | 20.2913 |
| **25** | Chatham County, GA | 13051 | Non-Hot Spot | Non-Hot Spot | Non-Hot Spot | 29.8517 |
| **26** | Chattahoochee County, | 13053 | Non-Hot Spot | Non-Hot Spot | Non-Hot Spot | 5.3247 |
| **27** | Chattooga County, GA | 13055 | Non-Hot Spot | Non-Hot Spot | Non-Hot Spot | 21.1292 |
| **28** | Cherokee County, GA | 13057 | Non-Hot Spot | Non-Hot Spot | Non-Hot Spot | 16.7997 |
| **29** | Clarke County, GA | 13059 | Non-Hot Spot | Non-Hot Spot | Non-Hot Spot | 17.4956 |
| **30** | Clay County, GA | 13061 | Non-Hot Spot | Non-Hot Spot | Non-Hot Spot | 58.9210 |
| **31** | Clayton County, GA | 13063 | Non-Hot Spot | Non-Hot Spot | Non-Hot Spot | 18.4684 |
| **32** | Clinch County, GA | 13065 | Non-Hot Spot | Non-Hot Spot | Non-Hot Spot | 32.9569 |
| **33** | Cobb County, GA | 13067 | Non-Hot Spot | Non-Hot Spot | Non-Hot Spot | 15.5999 |
| **34** | Coffee County, GA | 13069 | Non-Hot Spot | Non-Hot Spot | Non-Hot Spot | 27.8056 |
| **35** | Colquitt County, GA | 13071 | Non-Hot Spot | Non-Hot Spot | Non-Hot Spot | 29.6024 |
| **36** | Columbia County, GA | 13073 | Non-Hot Spot | Non-Hot Spot | Non-Hot Spot | 22.8200 |
| **37** | Cook County, GA | 13075 | Non-Hot Spot | Non-Hot Spot | Non-Hot Spot | 24.6528 |
| **38** | Coweta County, GA | 13077 | Non-Hot Spot | Non-Hot Spot | Non-Hot Spot | 24.4904 |
| **39** | Crawford County, GA | 13079 | Non-Hot Spot | Non-Hot Spot | Non-Hot Spot | 29.9033 |
| **40** | Crisp County, GA | 13081 | Non-Hot Spot | Non-Hot Spot | Non-Hot Spot | 28.2491 |
| **41** | Dade County, GA | 13083 | Non-Hot Spot | Non-Hot Spot | Non-Hot Spot | 28.5413 |
| **42** | Dawson County, GA | 13085 | Non-Hot Spot | Non-Hot Spot | Non-Hot Spot | 20.7932 |
| **43** | Decatur County, GA | 13087 | Non-Hot Spot | Non-Hot Spot | Non-Hot Spot | 36.4557 |
| **44** | DeKalb County, GA | 13089 | Non-Hot Spot | Non-Hot Spot | Non-Hot Spot | 21.7709 |
| **45** | Dodge County, GA | 13091 | Non-Hot Spot | Non-Hot Spot | Non-Hot Spot | 24.9478 |
| **46** | Dooly County, GA | 13093 | Non-Hot Spot | Non-Hot Spot | Non-Hot Spot | 23.8291 |
| **47** | Dougherty County, GA | 13095 | Non-Hot Spot | Non-Hot Spot | Non-Hot Spot | 32.6437 |
| **48** | Douglas County, GA | 13097 | Non-Hot Spot | Non-Hot Spot | Non-Hot Spot | 18.2147 |
| **49** | Early County, GA | 13099 | Non-Hot Spot | Non-Hot Spot | Non-Hot Spot | 57.1949 |
| **50** | Echols County, GA | 13101 | Non-Hot Spot | Non-Hot Spot | Non-Hot Spot | 17.7072 |
| **51** | Effingham County, GA | 13103 | Non-Hot Spot | Non-Hot Spot | Non-Hot Spot | 22.3128 |
| **52** | Elbert County, GA | 13105 | Non-Hot Spot | Hot Spot | Non-Hot Spot | 37.5505 |
| **53** | Emanuel County, GA | 13107 | Non-Hot Spot | Non-Hot Spot | Non-Hot Spot | 37.3248 |
| **54** | Evans County, GA | 13109 | Non-Hot Spot | Non-Hot Spot | Non-Hot Spot | 25.3538 |
| **55** | Fannin County, GA | 13111 | Non-Hot Spot | Non-Hot Spot | Non-Hot Spot | 30.1329 |
| **56** | Fayette County, GA | 13113 | Non-Hot Spot | Non-Hot Spot | Non-Hot Spot | 22.1062 |
| **57** | Floyd County, GA | 13115 | Non-Hot Spot | Non-Hot Spot | Non-Hot Spot | 19.5193 |
| **58** | Forsyth County, GA | 13117 | Non-Hot Spot | Non-Hot Spot | Non-Hot Spot | 14.6264 |
| **59** | Franklin County, GA | 13119 | Non-Hot Spot | Non-Hot Spot | Non-Hot Spot | 30.6706 |
| **60** | Fulton County, GA | 13121 | Non-Hot Spot | Non-Hot Spot | Non-Hot Spot | 24.8768 |
| **61** | Gilmer County, GA | 13123 | Non-Hot Spot | Non-Hot Spot | Non-Hot Spot | 25.8142 |
| **62** | Glascock County, GA | 13125 | Non-Hot Spot | Hot Spot | Non-Hot Spot | 27.9416 |
| **63** | Glynn County, GA | 13127 | Non-Hot Spot | Non-Hot Spot | Non-Hot Spot | 33.2780 |
| **64** | Gordon County, GA | 13129 | Non-Hot Spot | Non-Hot Spot | Non-Hot Spot | 19.9899 |
| **65** | Grady County, GA | 13131 | Non-Hot Spot | Non-Hot Spot | Non-Hot Spot | 33.7221 |
| **66** | Greene County, GA | 13133 | Non-Hot Spot | Non-Hot Spot | Non-Hot Spot | 40.9854 |
| **67** | Gwinnett County, GA | 13135 | Non-Hot Spot | Non-Hot Spot | Non-Hot Spot | 13.1469 |
| **68** | Habersham County, GA | 13137 | Non-Hot Spot | Non-Hot Spot | Hot Spot | 24.4705 |
| **69** | Hall County, GA | 13139 | Non-Hot Spot | Non-Hot Spot | Non-Hot Spot | 18.2582 |
| **70** | Hancock County, GA | 13141 | Non-Hot Spot | Non-Hot Spot | Non-Hot Spot | 37.4243 |
| **71** | Haralson County, GA | 13143 | Non-Hot Spot | Non-Hot Spot | Non-Hot Spot | 20.5138 |
| **72** | Harris County, GA | 13145 | Non-Hot Spot | Non-Hot Spot | Non-Hot Spot | 33.3829 |
| **73** | Hart County, GA | 13147 | Non-Hot Spot | Non-Hot Spot | Non-Hot Spot | 29.8028 |
| **74** | Heard County, GA | 13149 | Non-Hot Spot | Non-Hot Spot | Non-Hot Spot | 22.6526 |
| **75** | Henry County, GA | 13151 | Non-Hot Spot | Non-Hot Spot | Non-Hot Spot | 19.5522 |
| **76** | Houston County, GA | 13153 | Non-Hot Spot | Non-Hot Spot | Non-Hot Spot | 25.2922 |
| **77** | Irwin County, GA | 13155 | Non-Hot Spot | Non-Hot Spot | Non-Hot Spot | 30.5669 |
| **78** | Jackson County, GA | 13157 | Non-Hot Spot | Non-Hot Spot | Non-Hot Spot | 19.0663 |
| **79** | Jasper County, GA | 13159 | Non-Hot Spot | Non-Hot Spot | Non-Hot Spot | 29.8426 |
| **80** | Jeff Davis County, GA | 13161 | Non-Hot Spot | Non-Hot Spot | Non-Hot Spot | 30.9118 |
| **81** | Jefferson County, GA | 13163 | Hot Spot | Hot Spot | Non-Hot Spot | 43.3919 |
| **82** | Jenkins County, GA | 13165 | Hot Spot | Hot Spot | Hot Spot | 44.2798 |
| **83** | Johnson County, GA | 13167 | Non-Hot Spot | Non-Hot Spot | Non-Hot Spot | 29.5719 |
| **84** | Jones County, GA | 13169 | Non-Hot Spot | Non-Hot Spot | Non-Hot Spot | 29.9425 |
| **85** | Lamar County, GA | 13171 | Non-Hot Spot | Non-Hot Spot | Non-Hot Spot | 28.2984 |
| **86** | Lanier County, GA | 13173 | Non-Hot Spot | Non-Hot Spot | Non-Hot Spot | 25.0882 |
| **87** | Laurens County, GA | 13175 | Non-Hot Spot | Non-Hot Spot | Non-Hot Spot | 28.2808 |
| **88** | Lee County, GA | 13177 | Non-Hot Spot | Non-Hot Spot | Non-Hot Spot | 16.2786 |
| **89** | Liberty County, GA | 13179 | Non-Hot Spot | Non-Hot Spot | Non-Hot Spot | 15.2527 |
| **90** | Lincoln County, GA | 13181 | Non-Hot Spot | Hot Spot | Non-Hot Spot | 39.8080 |
| **91** | Long County, GA | 13183 | Non-Hot Spot | Non-Hot Spot | Non-Hot Spot | 18.4209 |
| **92** | Lowndes County, GA | 13185 | Non-Hot Spot | Non-Hot Spot | Non-Hot Spot | 23.1989 |
| **93** | Lumpkin County, GA | 13187 | Non-Hot Spot | Non-Hot Spot | Non-Hot Spot | 19.9165 |
| **94** | McDuffie County, GA | 13189 | Hot Spot | Hot Spot | Non-Hot Spot | 38.0006 |
| **95** | McIntosh County, GA | 13191 | Non-Hot Spot | Non-Hot Spot | Non-Hot Spot | 27.7191 |
| **96** | Macon County, GA | 13193 | Non-Hot Spot | Non-Hot Spot | Non-Hot Spot | 25.3110 |
| **97** | Madison County, GA | 13195 | Non-Hot Spot | Non-Hot Spot | Non-Hot Spot | 19.8180 |
| **98** | Marion County, GA | 13197 | Non-Hot Spot | Non-Hot Spot | Non-Hot Spot | 35.6030 |
| **99** | Meriwether County, GA | 13199 | Non-Hot Spot | Non-Hot Spot | Non-Hot Spot | 35.1561 |
| **100** | Miller County, GA | 13201 | Non-Hot Spot | Non-Hot Spot | Hot Spot | 41.5941 |
| **101** | Mitchell County, GA | 13205 | Non-Hot Spot | Non-Hot Spot | Non-Hot Spot | 32.6973 |
| **102** | Monroe County, GA | 13207 | Non-Hot Spot | Non-Hot Spot | Non-Hot Spot | 28.1587 |
| **103** | Montgomery County, GA | 13209 | Non-Hot Spot | Non-Hot Spot | Non-Hot Spot | 21.3133 |
| **104** | Morgan County, GA | 13211 | Non-Hot Spot | Non-Hot Spot | Non-Hot Spot | 32.4697 |
| **105** | Murray County, GA | 13213 | Non-Hot Spot | Non-Hot Spot | Non-Hot Spot | 16.2748 |
| **106** | Muscogee County, GA | 13215 | Non-Hot Spot | Non-Hot Spot | Non-Hot Spot | 30.6481 |
| **107** | Newton County, GA | 13217 | Non-Hot Spot | Non-Hot Spot | Non-Hot Spot | 24.5640 |
| **108** | Oconee County, GA | 13219 | Non-Hot Spot | Non-Hot Spot | Non-Hot Spot | 16.6018 |
| **109** | Oglethorpe County, GA | 13221 | Non-Hot Spot | Non-Hot Spot | Non-Hot Spot | 30.9100 |
| **110** | Paulding County, GA | 13223 | Non-Hot Spot | Non-Hot Spot | Non-Hot Spot | 13.7565 |
| **111** | Peach County, GA | 13225 | Non-Hot Spot | Non-Hot Spot | Non-Hot Spot | 28.8436 |
| **112** | Pickens County, GA | 13227 | Non-Hot Spot | Non-Hot Spot | Non-Hot Spot | 24.7076 |
| **113** | Pierce County, GA | 13229 | Non-Hot Spot | Non-Hot Spot | Non-Hot Spot | 26.3613 |
| **114** | Pike County, GA | 13231 | Non-Hot Spot | Non-Hot Spot | Non-Hot Spot | 31.5518 |
| **115** | Polk County, GA | 13233 | Non-Hot Spot | Non-Hot Spot | Non-Hot Spot | 20.2912 |
| **116** | Pulaski County, GA | 13235 | Non-Hot Spot | Non-Hot Spot | Non-Hot Spot | 29.4307 |
| **117** | Putnam County, GA | 13237 | Non-Hot Spot | Non-Hot Spot | Non-Hot Spot | 35.1872 |
| **118** | Quitman County, GA | 13239 | Non-Hot Spot | Non-Hot Spot | Non-Hot Spot | 15.8629 |
| **119** | Rabun County, GA | 13241 | Non-Hot Spot | Non-Hot Spot | Hot Spot | 33.7260 |
| **120** | Randolph County, GA | 13243 | Hot Spot | Non-Hot Spot | Non-Hot Spot | 46.7725 |
| **121** | Richmond County, GA | 13245 | Non-Hot Spot | Non-Hot Spot | Hot Spot | 33.6335 |
| **122** | Rockdale County, GA | 13247 | Non-Hot Spot | Non-Hot Spot | Non-Hot Spot | 23.8797 |
| **123** | Schley County, GA | 13249 | Non-Hot Spot | Non-Hot Spot | Non-Hot Spot | 16.6942 |
| **124** | Screven County, GA | 13251 | Hot Spot | Non-Hot Spot | Non-Hot Spot | 49.3735 |
| **125** | Seminole County, GA | 13253 | Hot Spot | Non-Hot Spot | Hot Spot | 44.9657 |
| **126** | Spalding County, GA | 13255 | Non-Hot Spot | Non-Hot Spot | Non-Hot Spot | 27.5986 |
| **127** | Stephens County, GA | 13257 | Non-Hot Spot | Non-Hot Spot | Non-Hot Spot | 35.6011 |
| **128** | Stewart County, GA | 13259 | Non-Hot Spot | Non-Hot Spot | Non-Hot Spot | 29.7164 |
| **129** | Sumter County, GA | 13261 | Non-Hot Spot | Non-Hot Spot | Non-Hot Spot | 41.2213 |
| **130** | Talbot County, GA | 13263 | Non-Hot Spot | Non-Hot Spot | Non-Hot Spot | 47.1422 |
| **131** | Taliaferro County, GA | 13265 | Non-Hot Spot | Non-Hot Spot | Non-Hot Spot | 29.4152 |
| **132** | Tattnall County, GA | 13267 | Non-Hot Spot | Non-Hot Spot | Non-Hot Spot | 21.3380 |
| **133** | Taylor County, GA | 13269 | Non-Hot Spot | Non-Hot Spot | Non-Hot Spot | 31.3151 |
| **134** | Telfair County, GA | 13271 | Non-Hot Spot | Non-Hot Spot | Non-Hot Spot | 27.1283 |
| **135** | Terrell County, GA | 13273 | Hot Spot | Non-Hot Spot | Non-Hot Spot | 44.8463 |
| **136** | Thomas County, GA | 13275 | Non-Hot Spot | Non-Hot Spot | Non-Hot Spot | 39.5530 |
| **137** | Tift County, GA | 13277 | Non-Hot Spot | Non-Hot Spot | Non-Hot Spot | 25.9193 |
| **138** | Toombs County, GA | 13279 | Non-Hot Spot | Non-Hot Spot | Non-Hot Spot | 37.2440 |
| **139** | Towns County, GA | 13281 | Non-Hot Spot | Non-Hot Spot | Hot Spot | 36.3728 |
| **140** | Treutlen County, GA | 13283 | Non-Hot Spot | Non-Hot Spot | Non-Hot Spot | 23.2475 |
| **141** | Troup County, GA | 13285 | Non-Hot Spot | Non-Hot Spot | Non-Hot Spot | 23.4665 |
| **142** | Turner County, GA | 13287 | Non-Hot Spot | Non-Hot Spot | Non-Hot Spot | 31.7809 |
| **143** | Twiggs County, GA | 13289 | Non-Hot Spot | Non-Hot Spot | Non-Hot Spot | 34.4216 |
| **144** | Union County, GA | 13291 | Non-Hot Spot | Non-Hot Spot | Non-Hot Spot | 34.9464 |
| **145** | Upson County, GA | 13293 | Non-Hot Spot | Hot Spot | Non-Hot Spot | 33.5945 |
| **146** | Walker County, GA | 13295 | Non-Hot Spot | Non-Hot Spot | Non-Hot Spot | 26.3520 |
| **147** | Walton County, GA | 13297 | Non-Hot Spot | Non-Hot Spot | Non-Hot Spot | 21.0443 |
| **148** | Ware County, GA | 13299 | Non-Hot Spot | Non-Hot Spot | Non-Hot Spot | 26.1655 |
| **149** | Warren County, GA | 13301 | Non-Hot Spot | Non-Hot Spot | Non-Hot Spot | 61.7230 |
| **150** | Washington County, GA | 13303 | Non-Hot Spot | Non-Hot Spot | Non-Hot Spot | 47.6404 |
| **151** | Wayne County, GA | 13305 | Non-Hot Spot | Non-Hot Spot | Non-Hot Spot | 26.2908 |
| **152** | Webster County, GA | 13307 | Hot Spot | Non-Hot Spot | Non-Hot Spot | 45.4729 |
| **153** | Wheeler County, GA | 13309 | Non-Hot Spot | Non-Hot Spot | Non-Hot Spot | 9.7822 |
| **154** | White County, GA | 13311 | Non-Hot Spot | Non-Hot Spot | Non-Hot Spot | 24.3296 |
| **155** | Whitfield County, GA | 13313 | Non-Hot Spot | Non-Hot Spot | Non-Hot Spot | 20.9779 |
| **156** | Wilcox County, GA | 13315 | Non-Hot Spot | Non-Hot Spot | Non-Hot Spot | 25.1717 |
| **157** | Wilkes County, GA | 13317 | Hot Spot | Hot Spot | Non-Hot Spot | 49.0857 |
| **158** | Wilkinson County, GA | 13319 | Non-Hot Spot | Non-Hot Spot | Non-Hot Spot | 36.3889 |
| **159** | Worth County, GA | 13321 | Non-Hot Spot | Non-Hot Spot | Non-Hot Spot | 29.6167 |
